# Food and nutrient intake at 1 year of age in Northern Sweden: results from the Swedish NICE birth cohort

**DOI:** 10.3389/fnut.2025.1548512

**Published:** 2025-02-13

**Authors:** Mia Stråvik, Mariza Kampouri, Klara Gustin, Anna Sandin, Agnes E. Wold, Malin Barman, Ann-Sofie Sandberg

**Affiliations:** ^1^Department of Life Sciences, Food and Nutrition Science, Chalmers University of Technology, Gothenburg, Sweden; ^2^Institute of Environmental Medicine, Karolinska Institutet, Stockholm, Sweden; ^3^Department of Clinical Science, Pediatrics, Sunderby Research Unit, Umeå University, Umeå, Sweden; ^4^Department of Infectious Diseases, Institute of Biomedicine, Sahlgrenska Academy, University of Gothenburg, Gothenburg, Sweden

**Keywords:** food intake, nutrients, children, cohort, micronutrients, dietary recommendations

## Abstract

Dietary habits and nutrient intake early in life are important for long-term health. Here, we examine food and nutrient intake at 1 year of age in the Swedish NICE (Nutritional impact on Immunological maturation during Childhood in relation to the Environment) birth cohort in relation to dietary guidelines and family characteristics. Dietary data was collected using a web-based semi-quantitative food frequency questionnaire (TodMeal-Q). Our findings show that intakes of critical micronutrients such as selenium, iodine, and iron were substantially below recommended levels. Also, the dietary patterns observed, characterized by higher protein and lower fat intake compared to recommendations, highlight the need for nutritional guidance to mitigate long-term health risks. Moreover, family dynamics, including the presence of siblings and maternal smoking habits, should be considered in designing effective dietary interventions, as these factors may be indicative of the context in which feeding practices are established.

## 1 Introduction

The first years of life are critical for development and establishing the foundations of long-term health. Nutrition during this period plays a crucial role in supporting fundamental aspects such as physical growth, cognitive development, and immune function, and is important for preventing a wide range of non-communicable diseases (1–3).

The World Health Organization (WHO) has identified malnutrition in young children as a global health challenge (4). Although severe malnutrition due to energy deficiency is rare in high-income countries like Sweden, deficiencies in essential micronutrients can still occur even when overall energy intake is sufficient. In Sweden, growing concerns have emerged regarding low intakes of critical nutrients, including iodine, iron, and vitamin D (5–8). These shortage of nutrients may have serious long-term implications for children’s health and development (9–11), underscoring the need for comprehensive studies on nutrient intake and dietary patterns among Swedish children.

Most dietary guidelines, including those in Nordic countries, emphasize the importance of consuming a diet rich in fruits, vegetables, and whole grains while prioritizing unsaturated fats and fish over saturated fats and other animal-based meats (12). However, data regarding adherence to these guidelines remains limited, particularly for young children. Despite the well-documented importance of early-life nutrition, few studies have comprehensively investigated young children’s food and nutrient intake in Sweden. The Swedish Food Agency has explicitly highlighted the need for nationally representative data on dietary habits of children under 4 years of age (13). Their most recent dietary survey investigated food intake among Swedish children but their report did not provide any information about one-year-olds (7).

Therefore, this study aims to provide a comprehensive analysis of children’s food and nutrient intake in Sweden at 1 year of age, utilizing a prospective birth cohort established in Northern Sweden. In previous research within this cohort, we demonstrated that many mothers have inadequate intakes of several nutrients during pregnancy and postpartum (14, 15), with these intakes significantly varying based on socioeconomic factors (15). Hence, an additional aim of this study was to examine whether children’s dietary intakes align with current dietary guidelines and to explore how family characteristics influence these nutritional patterns.

## 2 Materials and methods

### 2.1 Study design

The NICE study (ClinicalTrials.gov identifier: NCT05809479) recruited pregnant women and their partners at a routine ultrasound during gestational week 18. The potential study participants received written information about the study and signed written informed consent for themselves and their children before being enrolled. All expecting parents living within the catchment area of Sunderby Hospital in Norrbotten County and with the ability to communicate in Swedish were invited to participate. The recruitment took place from February 2015 to March 2018, and 655 families were successfully recruited. More detailed information about the study can be found in the study protocol (16). The NICE study was approved by the Regional Ethical Review Board in Umeå, Sweden (2013/18-31 M, 2015–71–32) and conducted following the Helsinki Declaration.

#### 2.1.1 Inclusion and exclusion criteria

At 1 year of age, parents of the 598 infants who were still in the study (9% were lost to follow-up) received a link to the food frequency questionnaire, and 538 families responded. Out of the 538 responses, we excluded twins and the second child in cases where the family participated with multiple infants. Therefore, a total of 523 children with dietary data were eligible and included in this study.

By 1 year of age, some infants consume both solid food and breast milk or infant formula. For this study’s primary analyses, we focused exclusively on children who had stopped receiving breast milk and infant formula by 10 months of age. This decision was made since information on the amount and frequency of breast milk and formula intake were lacking. Including these children could lead to an overestimation of the proportion of children with inadequate nutrient intake from dietary sources, as the nutritional needs of the breastfed or formula-fed children might be partly met by breast milk or infant formula. Initially, 523 children were considered, but several exclusion criteria narrowed down the sample size. First, 149 children were excluded because they were still breastfeeding during the collection period. An additional 10 children were excluded due to insufficient breastfeeding duration information. After these initial exclusions, 364 children remained in the potential sample. Of these, 95 were further excluded for using infant formula during the collection period, and an additional 18 were removed due to incomplete formula use information. Ultimately, the primary analyses included 251 children who had discontinued both breast milk and formula by 10 months of age. A detailed exclusion flow chart is available in Supplementary Figure S1.

### 2.2 Food frequency questionnaire

When the child reached 1 year of age (mean (range): 365 (357–400) days), parents received a web-based semiquantitative food frequency questionnaire (TodMeal-Q). The parents were instructed to report what the child usually ate and drank at home, daycare, and other places, considering both weekdays and weekends over the past few months. Parents were asked to respond only if the child consumed the food and to skip questions about rarely or never consumed foods. Since missing values represent a lack of consumption, they were coded as zero instead of missing.

The questionnaire comprised 19–41 questions, depending on the number of follow-up questions. Each question covered several food items. For example, parents were asked to indicate how often their child consumed beverages such as water, milk, plant-based milk alternatives, juice, soda, and gruel, with frequency options ranging from 1–3 times/month to 3 times/day or more. If a food item was reported (e.g., yoghurt), a follow-up question specified the type (e.g., natural or flavored). For bread consumption, two follow-up questions were asked: (1) the number of slices consumed per occasion (ranging from less than one slice to seven or more), and (2) the type of spreadable fat used.

To further quantify the child’s food intake, three questions regarding portion sizes were included: (1) “How much meat, fish, or vegetarian alternatives does your child usually eat?” (2) “How much potato, rice, pasta, or similar does your child usually eat?” and (3) “How much raw or cooked vegetables does your child usually eat?”. Respondents chose from five pictures depicting different portion sizes for each question, with each picture corresponding to a specific intake in grams. For meat, fish, or vegetarian alternatives, the pictures showed a plate with meatballs. For potato, rice, and pasta, the pictures showed a plate with pasta (fusilli). For vegetables, the pictures showed a plate with a mix of sliced cucumber, lettuce, corn, and tomato.

Parents also specified the amount of chocolate, candy, or seasoned nuts consumed (a few pieces, 50 g, 100 g or more, do not know), ice cream (less than 1 scoop, 1 scoop, 2 scoops, 3 scoops or more, do not know), and chips, popcorn, or cheese doodles consumed (a few pieces, 50 g, 100 g or more, do not know).

Based on the reported amounts and intake frequency, variables were created to represent intakes in grams per day. For food items not covered by the pictures or the specified amount questions, portion sizes were estimated manually by first author M.S. using a combination of kitchen scale (Soehnle no: 66160; Precision: 1 g), information from the Swedish Food Agency’s database (17), and the nutritional calculation software Dietist Net (18). All assumptions and estimations for quantifying the dietary data are presented in Supplementary Table S1.

#### 2.2.1 Estimation of energy needs

The estimated basal need (i.e., average resting energy expenditure, REE) was calculated using the Henry equation (19). It was calculated in megajoules (MJ) per day using gender, and parent-reported weight and height and converted to kilocalories (kcal) per day using the conversion factor of 239 (1 MJ = 239 kcal):

$$\mathrm{Boys}:\left(\left(0.118\times \mathrm{weight}\right)+\left(3.59\times \mathrm{height}\right)-1.55\right)\times 239$$

$$\mathrm{Girls}:\left(\left(0.127\times \mathrm{weight}\right)+\left(2.94\times \mathrm{height}\right)-1.20\right)\times 239$$

The average energy requirement of the children was calculated in kilojoule (kJ) per kilogram bodyweight and converted to kcal per day using the conversion factor 4.184 (1 kcal = 4.184 kJ):

$$\mathrm{Boys}:\frac{337\times \mathrm{weight}}{4.184}\;\mathrm{Girls}:\frac{333\times \mathrm{weight}}{4.184}$$

The food intake level (FIL) was estimated to evaluate the reported energy intake and calculated as a ratio between the reported energy intake and the calculated basal need (REE).

### 2.3 Family characteristics

At recruitment the parents received an e-mail with a link to a web-based questionnaire capturing family characteristics such as education level, tobacco use and type of animals at home. A total of 116–159 questions were included, depending on the number of follow-up questions. Furthermore, at 18 months after the child was born, the parents received one questionnaire each covering questions regarding profession, income and home environment.

### 2.4 Data analysis

IBM SPSS ver. 28 (IBM, New York, NY, United States) and R ver. 4.3.2 (Vienna, Austria) software packages were used for the data analysis. *p*-values were adjusted for multiple comparisons using the *p.adjust* function in R (20), applying the false discovery rate (FDR) method by Benjamini and Hochberg. Briefly, this method ranks each *p*-value after arranging them in ascending order, then multiplies each *p*-value by the total number of tests and divides the result by the specific rank. Here, the total number of tests was set based on the full set of diet variables, not solely the ones visualized in the result section, to give a more accurate result. Correlation coefficients were visually displayed with heatmaps using the *pheatmap* package in R (21), and significant associations with categorical outcome variables were further investigated with Mann–Whitney *U* (binary) or Kruskal–Wallis (multilevel) test. When comparing categorical variables between two groups (e.g., breastfed versus non-breastfed), the Chi-Square test, Linear-by-Linear Association test, and Fisher’s Exact test were used. Visualization of whether the children met the recommended intake levels of the nutrients was made with the *likert* package in R (22). All code for the statistical analyses is available at GitLab: https://gitlab.com/miastravik/.

The family characteristics investigated in relation to the child’s food intake were chosen *a priori* based on previous literature and reasoning of plausible factors which could be related to the feeding of a child. The following variables were included and the coding of each variable is presented from low to high (e.g., “no, yes” where no = 0 and yes = 1): age of the mother at the time of delivery (years, continuous), maternal education registered at the delivery ward [years, categorical: 9 years (e.g., elementary school), 12 years (e.g., high school), more than 12 years (e.g., university or other higher education), maternal BMI at admission to maternity ward (kg/m^2^, continuous)], number of siblings assessed by maternal parity at delivery ward (number of previous births, continuous), income of both mother and father as reported in individual questionnaires sent out 18 months after the delivery (categorical, yearly income before taxes, including, e.g., child support and unemployment insurance: 0 SEK, <150.000 SEK, 150.000–199.999 SEK, 200.000–299.999 SEK, 300.000–399.999 SEK, 400.000–499.999 SEK, >500.000 SEK), Swedish nationality from the mother’s side as reported to the delivery ward (categorical: not Swedish, Swedish), allergic heredity from either mother, father or siblings as reported at the 1 year visit (categorical: no, yes); pet at home assessed at 1 year visit (categorical: no, yes), area of living as reported at 1 year visit (categorical: city, village or smaller town, house in the countryside, farm with animals), maternal smoking before pregnancy as reported to the delivery ward (categorical: no, yes), and gender of the child as reported by the delivery ward (categorical: girl, boy).

## 3 Results

### 3.1 Energy parameters of the children

Among the 251 children who relied solely on dietary sources to meet their nutritional demands at 1 year of age, the median reported energy intake was 806 kcal with the lowest reported intake being 144 kcal and the highest 2,801 kcal. The basal energy need ranged from 421 to 841 kcal. As can be seen in Table 1, the average REE was higher among boys (median: 589 kcal) than girls (median: 544 kcal) at 1 year of age, although the variation was higher among boys. Following this pattern, the reported energy intake was slightly higher among boys (median: 821 kcal) than girls (median: 798 kcal). The corresponding data for all children, regardless of breastfeeding and formula feeding, are presented in Supplementary Table S2. Briefly, children who were still breast-or formula-fed at the time of the dietary assessment (i.e., 10 months of age or later, *N* = 272) had significantly lower energy intake from dietary sources than children who were not given breast milk or formula at the time (*N* = 251) (median (25th–75th percentile): 703 (548–869) versus 806 (681–938) kcal, *p* < 0.001).

**Table 1:** Energy intake and estimated needs of the children who were not fed breast milk or formula.

Variable	*N*	Mean (SD)	Median (IQR)	Min–max
All children (*N* = 251)				
Energy intake (kcal)	251	832.02 (265.83)	805.59 (252.08)	144.38–2801.14
Average Energy Requirement^1^	249	805.61 (98.07)	805.45 (161.09)	557.12-11π27.63
Resting Energy Expenditure^2^	246	561.75 (58.81)	555.08 (75.96)	421.45–840.68
Food Intake Level^3^	246	1.49 (0.46)	1.4 (0.51)	0.26–4.1
Height (m)	246	0.76 (0.04)	0.76 (0.04)	0.61–1.05
Weight (kg)	249	10.06 (1.2)	10 (2)	7–14
Boys (*N* = 124)				
Energy intake (kcal)	124	849.6 (317.07)	820.91 (271.84)	144.38–2801.14
Average Energy Requirement	123	840.15 (94)	805.45 (80.54)	563.81–1127.63
Resting Energy Expenditure	121	587.67 (59.72)	589.4 (73.56)	421.45–840.68
Food Intake Level	121	1.45 (0.52)	1.38 (0.52)	0.26–4.1
Height (m)	121	0.77 (0.04)	0.78 (0.05)	0.61–1.05
Weight (kg)	123	10.43 (1.17)	10 (1)	7–14
Girls (*N* = 127)				
Energy intake (kcal)	127	814.86 (203.65)	797.59 (214.67)	174.54–1606.84
Average Energy Requirement	126	771.89 (90.2)	795.89 (79.59)	557.12–1034.66
Resting Energy Expenditure	125	536.65 (45.75)	543.73 (58.45)	433.83–653.62
Food Intake Level	125	1.52 (0.4)	1.45 (0.45)	0.32–2.88
Height (m)	125	0.75 (0.03)	0.75 (0.03)	0.68–0.83
Weight (kg)	126	9.7 (1.13)	10 (1)	7–13

^1^Based on Table 6 in the NNR Energy Paper (37). In kJ per kg bodyweight. Boys: (337*weight)/4.184; Girls: (333*weight)/4.184.

^2^Based on Table 5 in the NNR Energy Paper (37). Calculates only the basal energy need (i.e., not considering physical movement). Boys: ((0.118*weight) + (3.59*height_m)-1.55) *239; Girls: ((0.127*weight) + (2.94*height_m)-1.20) *239.

^3^Food intake level (FIL) = reported energy intake divided by predicted basal metabolic rate (kcal/REE).

### 3.2 Intake of food groups and food items

The intakes of food items and food groups are presented in Table 2 (children who were not breast-or formula-fed at the time) and in Supplementary Table S3 (all children regardless of breast-and formula feeding). Gruel (“välling”), primarily in the form of ready-to-blend powder, was the most consumed food group at this age. Regarding other carbohydrate sources, pasta and potatoes were most commonly consumed while rice and, e.g., bulgur were not consumed to the same extent. Bread was consumed almost exclusively as refined varieties (i.e., not whole grain varieties).

**Table 2:** Intake of food items among the children who did not receive breast milk or formula.

Food item	*N*	Mean (SD)	Median (IQR)	Min–max
Bulgur, couscous or quinoa	251	4.53 (10.13)	0 (5.36)	0–110
Rice	251	16.49 (14.62)	16.07 (18.21)	0–110
Pasta or noodles	251	35.67 (23.67)	37.5 (38.93)	0–176.79
Total potato	251	40.66 (26.11)	37.5 (38.93)	0–196.43
French fries or fried potato	251	5.18 (9.18)	0 (5.36)	0–86.43
Boiled or mashed potato	251	35.48 (23.9)	37.5 (38.93)	0–150
Vegetarian meat alternatives	251	7.16 (13.86)	0 (6.57)	0–72.29
Beans or lentils	251	1.02 (4.71)	0 (0)	0–36.14
Soy products	251	0.44 (3.16)	0 (0)	0–36.14
Quorn	251	0.36 (2.55)	0 (0)	0–24.1
Roots	251	1.39 (6.06)	0 (0)	0–46
Total seafood	251	20.81 (16.69)	19.71 (19.93)	0–92
Seafood (without caviar)	251	20.78 (16.69)	19.71 (19.93)	0–92
Lean Fish	251	10.92 (12.47)	9.07 (14.5)	0–92
Fish fingers	251	2.58 (5.63)	0 (3.11)	0–46
Cod or pollock	251	7.78 (10.77)	6.21 (9.86)	0–92
Tuna	251	0.57 (2.71)	0 (0)	0–23
Fatty fish	251	7.82 (9.29)	6.21 (9.86)	0–63.5
Shellfish	251	0.79 (2.65)	0 (0)	0–21.17
Other fish	251	0.28 (1.92)	0 (0)	0–23
Total vegetables	251	39.23 (43.42)	23.57 (52.5)	0–270
Tomato or bell pepper	251	4.68 (7.68)	1.67 (7.5)	0–67.5
Cucumber or lettuce	251	3.85 (7.03)	1.07 (5.76)	0–67.5
Carrot	251	6.32 (8.95)	3 (8.39)	0–67.5
Onion	251	1.97 (4.32)	0 (1.67)	0–30
Corn	251	5.63 (8.4)	2.95 (7.86)	0–67.5
Avocado	251	1.93 (4.37)	0 (1.64)	0–30
Cauliflower or cabbage	251	1.35 (4.58)	0 (0)	0–40
Broccoli	251	3.9 (6.01)	0.64 (6)	0–30
Spinach	251	0.2 (1.16)	0 (0)	0–10
Green peas	251	4.97 (7.88)	1.5 (7.68)	0–60
Beans, lentils or chickpeas	251	1.55 (4.36)	0 (0)	0–40
Parsnip, Swedish turnip or celeriac	251	2.34 (6.11)	0 (0)	0–45
Pickled vegetables	251	0.1 (1.06)	0 (0)	0–13.33
Other vegetables	251	0.41 (2.43)	0 (0)	0–24
Water	251	534.89 (134.45)	600 (0)	0–600
Sugary drinks	251	10.93 (61.21)	0 (0)	0–642.86
Total soda	251	6.26 (53.68)	0 (0)	0–600
Sugar	251	3.59 (42.27)	0 (0)	0–600
Artificial sweeteners	251	1.2 (18.94)	0 (0)	0–300
Juice	251	4.67 (24.96)	0 (0)	0–200
Plant-based dairy alternatives	251	11.5 (55.29)	0 (0)	0–600
Oat drink	251	9.76 (54.77)	0 (0)	0–600
Soy drink	251	0 (0)	0 (0)	0–0
Sesame drink	251	0 (0)	0 (0)	0–0
Coconut milk	251	0.26 (4.06)	0 (0)	0–64.29
Yoghurt	251	50.6 (78.19)	14.29 (42.86)	0–400
Plain	251	23.35 (58)	0 (0)	0–400
Flavored	251	12.88 (53.9)	0 (0)	0–400
Other yoghurt	251	2.98 (17.26)	0 (0)	0–128.57
Soup	251	4.95 (10.95)	0 (0)	0–42.86
Total gruel (“välling”)	251	275.52 (204.3)	225 (433.93)	0–675
Mild whole grain	251	112.44 (174.83)	0 (225)	0–675
Whole grain	251	129.92 (181.5)	0 (225)	0–675
Corn gruel or gluten-free	251	10.01 (56.38)	0 (0)	0–675
Other gruel	251	21.17 (89.76)	0 (0)	0–675
Porridge or rice pudding	251	142.94 (114.1)	130 (242.5)	0–425
Oatmeal	251	42.16 (84.6)	0 (64.29)	0–400
Semolina or rice	251	7.01 (29.19)	0 (0)	0–225
Snack, e.g., rice pudding	251	0 (0)	0 (0)	0–0
Powder-based for child	251	88.89 (95.02)	65 (130)	0–390
Other porridge	251	4.87 (30.9)	0 (0)	0–365
Nuts	251	0.1 (0.69)	0 (0)	0–9.64
Raisins	251	2.12 (6.9)	0 (0)	0–42
Egg	251	8.39 (11.69)	3.57 (10.71)	0–50
Bread and crackers	251	32.53 (35.11)	20 (40)	0–180
Total bread	251	31.84 (34.81)	20 (39.29)	0–180
Total wholegrain bread	251	9.11 (19.12)	0 (12.86)	0–124.02
Soft wholegrain bread	251	8.07 (17.81)	0 (4.29)	0–120
Crispbread	251	1.04 (3.49)	0 (0)	0–18.75
White bread	251	22.73 (27.16)	12.86 (38.57)	0–120
Rusks, rice cakes, or digestive biscuits	251	0.7 (2.42)	0 (0)	0–15
Sandwich toppings				
Cheese	251	3.89 (6.43)	0 (7.23)	0–45
Ham	251	1.48 (3.7)	0 (0)	0–22.5
Marmalade	251	0 (0)	0 (0)	0–0
Liver pâte	251	2.39 (4.82)	0 (2.5)	0–22.5
Caviar	251	0.04 (0.39)	0 (0)	0–5
Messmör (whey cheese)	251	0.11 (0.85)	0 (0)	0–9
Tomato	251	0.73 (3.82)	0 (0)	0–30
Cucumber	251	2.09 (6.57)	0 (0)	0–45
Total breakfast cereals	251	0.73 (4.41)	0 (0)	0–32.5
Cornflakes or Special K	251	0.03 (0.41)	0 (0)	0–6.43
Musli or wholegrain cereals (e.g., All bran)	251	0.2 (2.29)	0 (0)	0–25.71
Oat pillows or Cheerios	251	0.26 (2.7)	0 (0)	0–30
Sweet cereals (e.g., Frosties)	251	0.03 (0.41)	0 (0)	0–6.43
Other breakfast cereals	251	0.21 (2.43)	0 (0)	0–32.5
Total fruit and berries	251	163.62 (96.62)	190 (122.36)	0–495.71
Jam or applesauce	251	1.71 (5.42)	0 (0)	0–40
Sweetened fruit soup	251	4.78 (28.25)	0 (0)	0–200
Total fruit and berries (without juice)	251	152.46 (90.16)	167.5 (114.91)	0–345
Banana	251	44.3 (35.78)	35 (49)	0–210
Apple	251	45.62 (38.84)	41.67 (75)	0–187.5
Orange	251	20.01 (29.36)	0 (41.67)	0–125
Berries	251	18.71 (18.37)	15.63 (31.25)	0–93.75
Kiwi	251	6.85 (16.05)	0 (0)	0–85
Grapes	251	6.07 (16.8)	0 (0)	0–93.75
Other fruit	251	10.88 (29.58)	0 (0)	0–315
Total sauce	251	0.96 (3.04)	0 (0)	0–15
Vinaigrette	251	0.01 (0.15)	0 (0)	0–2.36
Ketchup	251	0.19 (0.95)	0 (0)	0–7.5
Creme fraiche	251	0.54 (2.02)	0 (0)	0–15
Bearnaise	251	0.09 (0.64)	0 (0)	0–5.89
Other sauce	251	0.14 (0.98)	0 (0)	0–10.02
Pancake	251	9.88 (11.44)	10 (10)	0–70
Pizza	251	3 (5.58)	0 (9.64)	0–28.93
Lasagna	251	7.06 (9.9)	0 (16.07)	0–37.5
Dairy products^1^	251	114.9 (153.32)	46.07 (166.07)	0–1,000
Cow’s milk	251	59.76 (115.53)	0 (42.86)	0–600
Total meat	251	76.67 (44.52)	70.43 (59.76)	0–254
Cured meat	251	22.69 (23.74)	19.66 (22.43)	0–205.5
Red meat	251	61.66 (38.21)	58 (51.48)	0–232.71
Offal^2^	251	7.32 (10.62)	4.14 (10.79)	0–71
Black pudding	251	4.93 (7.94)	0 (6.57)	0–63.5
Sausage	251	13.88 (15.65)	12.43 (19.71)	0–127
Meat dish	251	10.84 (14.94)	4.14 (19.71)	0–127
Chicken	251	15.01 (13.85)	12.43 (15.57)	0–99.79
Hamburger	251	2.77 (4.73)	0 (5.36)	0–27.21
Minced meat	251	25.36 (19.79)	19.71 (16.57)	0–127
Total root vegetables	251	47.5 (34.45)	40.71 (44.27)	0–200
Total without onion	251	45.53 (33.05)	40 (42.32)	0–200
Total without onion and potato	251	10.05 (14.97)	3.93 (11.86)	0–94.5
Buns, biscuits, or cookies	251	1.04 (3.67)	0 (1.21)	0–51
Candy	251	0.09 (0.9)	0 (0)	0–12.86
Chips	251	0.01 (0.15)	0 (0)	0–1.71
Ice cream	251	0.21 (1.14)	0 (0)	0–11.25
Probiotics	251	5.16 (23.38)	0 (0)	0–165

^1^Includes reported intake of cow’s milk (in glass or plate), yoghurt, whey cheese (“Messmör”), Cheese, and Créme fraiche.

^2Includes liver paste and black pudding.^

Regarding food groups commonly referred to as protein sources (e.g., meat and fish), meat was consumed primarily as dishes made of minced meat, chicken, and sausages. Also, a quite common intake in this group of children was liver pâté (“leverpastej”) as a spreadable meat topping on their bread (31% of the children had a regular consumption). Plant-based meat alternatives were consumed to some extent by 40% of the children, although the general intake was low. Fish and shellfish were most commonly consumed 1–2 times/week (49% of the children), followed by 3–4 times/week (22% of the children), and 1–3 times/month (18% of the children). To note, the children had a 3.5 times higher intake of meat than of fish and shellfish.

Regarding dairy products, cow’s milk as a beverage or on a plate was consumed at least once a month by 49% of the children. Among the cow’s milk consumers, 33% (16% of all children) consumed it 1–3 times/month, 21% (10% of all children) consumed it 1–2 times/week, and 31% (15% of all children) consumed it daily. Yoghurt was consumed at least once a month by 60% of all children and most commonly consumed 1–2 times/week.

Vegetable intake was generally low with only 38% of the children consuming it daily. To note, 16 children (6%) did not eat vegetables at all or consumed it more rarely than once a month. Fruits and berries, on the other hand, was the most consumed food group after gruel, with 77% of the children consuming it daily. To note, 5% did not consume fruit and berries at all or consumed it more rarely than once a month. The most common frequently consumed fruit was banana followed by apples or pears, although the estimated median intake was higher for apples and pears than for bananas.

Confectioneries, such as candy, chips, ice cream, and soda, were rarely consumed at this age. For instance, candy (including chocolate and seasoned nuts) was given to 13 children out of which 10 consumed it one to three times per month, two consumed it one to two times a week, and one child received it three to six times a week.

### 3.3 Nutrient composition

In Table 3, the intake of micro-and macronutrients calculated based on the whole diet is shown for the 251 children, and in Supplementary Table S4, the corresponding data are presented for all the 523 children regardless of breast-and formula feeding. To note, children who were excluded from the main analyses due to breast-or formula feeding had significantly lower reported intakes of all nutrients from dietary sources, except for monosaccharides, sucrose, fatty acid 4:0–10:0, fatty acid 20:0, beta-carotene, vitamin K, iodine, and trans fatty acids, where the differences were not statistically significant.

**Table 3:** Nutrient intake from the whole diet of children who did not receive breastmilk or formula.

Variable	*N*	Mean (SD)	Median (IQR)	Min–max
Energy (kcal)	251	832.02 (265.83)	805.59 (252.08)	144.38–2801.14
Protein (g)	251	34.09 (10.16)	33.21 (11.95)	4.96–72.17
Fat (g)	251	27.45 (11.09)	25.91 (12.12)	3.98–123.58
Carbohydrates (g)	251	106.23 (34.57)	102.37 (30.08)	19.44–336.01
Fiber (g)	251	9.92 (4.08)	9.28 (4.52)	2.21–40.67
Salt (g)	251	3.15 (1.27)	2.91 (1.61)	0.32–8.46
Water (g)	251	1089.36 (232.47)	1077.22 (263.59)	451.43–2028.21
Monosaccharides (g)	251	15.76 (8.64)	14.57 (10.37)	0.92–76.3
Disaccharides (g)	251	29.99 (12.79)	28.45 (13.7)	1.03–105.65
Sucrose (g)	251	10.28 (5.02)	9.48 (4.59)	0.73–39.4
Whole grain (g)	251	102.43 (54.89)	93.49 (84.39)	5.83–249.09
Sum Saturated fatty acids (g)	251	10.85 (4.17)	10.53 (5.22)	0.91–28.37
Fatty acid 4:0–10:0 (g)	251	0.54 (0.42)	0.45 (0.6)	0.01–2.16
Fatty acid 12:0 (g)	251	0.33 (0.2)	0.3 (0.21)	0.02–1.49
Fatty acid 14:0 (g)	251	0.93 (0.5)	0.82 (0.69)	0.08–2.81
Fatty acid 16:0 (g)	251	6.9 (2.36)	6.74 (2.91)	0.6–15.8
Fatty acid 18:0 (g)	251	1.92 (0.88)	1.78 (1.1)	0.13–8.05
Fatty acid 20:0 (g)	251	0.05 (0.04)	0.05 (0.04)	0–0.5
Sum Monounsaturated fatty acids (g)	251	10.24 (5.4)	9.61 (4.7)	1.61–72.77
Fatty acid 16:1 (g)	251	0.44 (0.22)	0.41 (0.29)	0.01–1.49
Fatty acid 18:1 (g)	251	9.5 (5.16)	8.92 (4.33)	1.35–70.69
Sum Polyunsaturated fatty acids (g)	251	4.27 (1.45)	4.05 (1.76)	0.97–13.85
Fatty acid 18:2 (g)	251	3.55 (1.25)	3.41 (1.42)	0.52–12.29
Fatty acid 20:4 (g)	251	0.04 (0.02)	0.04 (0.03)	0–0.15
Fatty acid 18:3 (g)	251	0.41 (0.21)	0.36 (0.22)	0.08–1.25
EPA (Fatty acid 20:5) (g)	251	0.05 (0.05)	0.03 (0.05)	0–0.37
DPA (Fatty acid 22:5) (g)	251	0.03 (0.02)	0.02 (0.02)	0–0.2
DHA (Fatty acid 22:6) (g)	251	0.11 (0.09)	0.08 (0.1)	0–0.68
Cholesterol (mg)	251	94.25 (47.9)	86.52 (56.05)	2.36–298.83
Vitamin A (μg)	251	426.91 (175.59)	412.69 (215.96)	40.81–1046.51
Retinol (μg)	251	351.08 (159.94)	344.88 (176.45)	14.4–908.53
Beta-Carotene (μg)	251	778.47 (858.49)	483.29 (824.19)	26.15–6412.19
Vitamin D (μg)	251	8.23 (3.52)	8.32 (3.96)	0.48–22.03
Vitamin E (mg)	251	8.39 (3.51)	8.3 (4.5)	0.88–24.37
Vitamin K (μg)	251	12.33 (9.6)	9.36 (11.55)	0.27–44.99
Thiamine (mg)	251	0.71 (0.2)	0.69 (0.23)	0.09–1.51
Riboflavin (mg)	251	0.83 (0.3)	0.8 (0.35)	0.1–2.34
Vitamin C (mg)	251	76.17 (32.25)	78.01 (47.58)	4.9–158.92
Niacin (mg)	251	7.41 (2.49)	7.16 (2.9)	1.22–17.58
Niacin equivalents (mg)	251	13.64 (4.18)	13.28 (5.28)	2.05–31.86
Vitamin B6 (mg)	251	0.91 (0.29)	0.89 (0.31)	0.22–3.07
Vitamin B12 (μg)	251	2.36 (0.98)	2.21 (1.22)	0.26–6.59
Phosphorus (mg)	251	714.34 (223.29)	684.31 (244.93)	131.89–1696.67
Folate (μg)	251	113.99 (45.52)	109.34 (61.3)	23.86–272.4
Iodine (μg)	251	48.34 (46.91)	30.41 (46.46)	1.02–352.11
Iron (mg)	251	9.86 (3.52)	9.63 (4.28)	1.09–21.11
Calcium (mg)	251	629.11 (256.65)	598.82 (296.73)	52.56–1973.82
Potassium (mg)	251	1420.57 (443.57)	1393.16 (511.59)	301.16–3413.48
Magnesium (mg)	251	140.94 (41.9)	137.82 (42.09)	31.65–394.92
Sodium (mg)	251	1247.09 (508.2)	1149.42 (643.08)	119.6–3381.11
Selenium (μg)	251	14.16 (5.79)	13.12 (7.31)	1.42–42.43
Zinc (mg)	251	4.59 (1.27)	4.52 (1.77)	0.78–8.68
Sugars (g)	251	2.17 (4.18)	0.07 (1.54)	0–21.6
Sum trans fatty acids (g)	251	0.16 (0.16)	0.11 (0.18)	0–0.98

#### 3.3.1 Macronutrient intake in relation to recommendations

**Figure 1:**
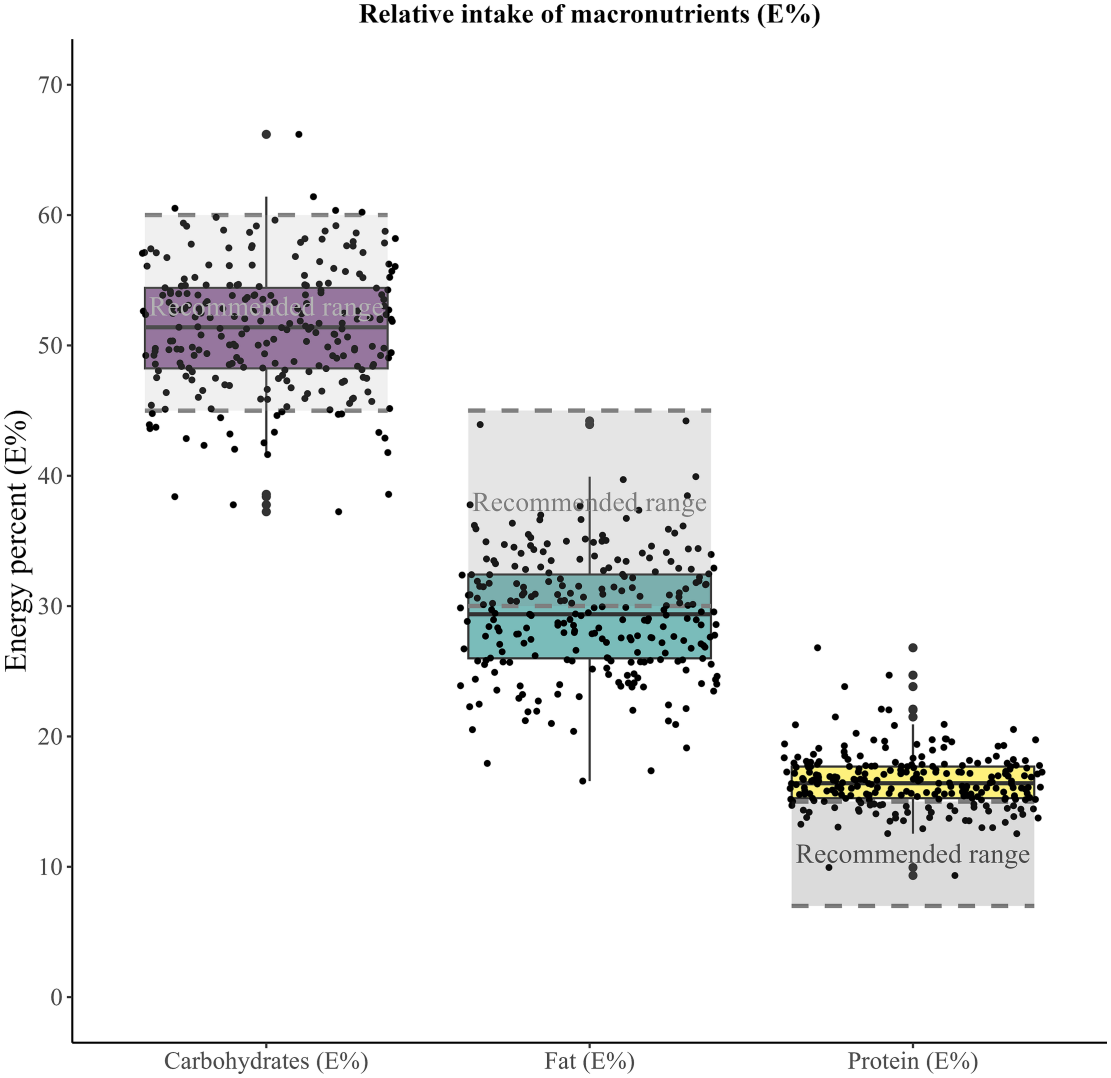
Macronutrient intakes of the 251 children receiving only food (breastfed and formula-fed children at 10 months of age excluded) in relation to the recommended ranges. The recommended ranges are those given in the Nordic Nutrition Recommendations (NNR) for 11 months old children: Carbohydrates, 45–60 E%; Fat, 30–45 E%; Protein, 7–15 E%.

Figure 1 displays the relative intake of carbohydrates, fat, and protein. The children’s average intake falls within the recommended ranges for carbohydrates (reported 51 E% versus recommended 45–60 E%), but not for fat (29 E% versus 30–45 E%) or protein (17 E% versus 7–15 E%). In more detail, 137 (55%) of the children had a relative fat intake below the recommended intake, while none consumed more than the recommended intake. Regarding protein, none of the children had an intake below the recommendation, while 203 (81%) had a relative intake exceeding the recommendation.

##### 3.3.1.1 Intake of fatty acids

**Figure 2:**
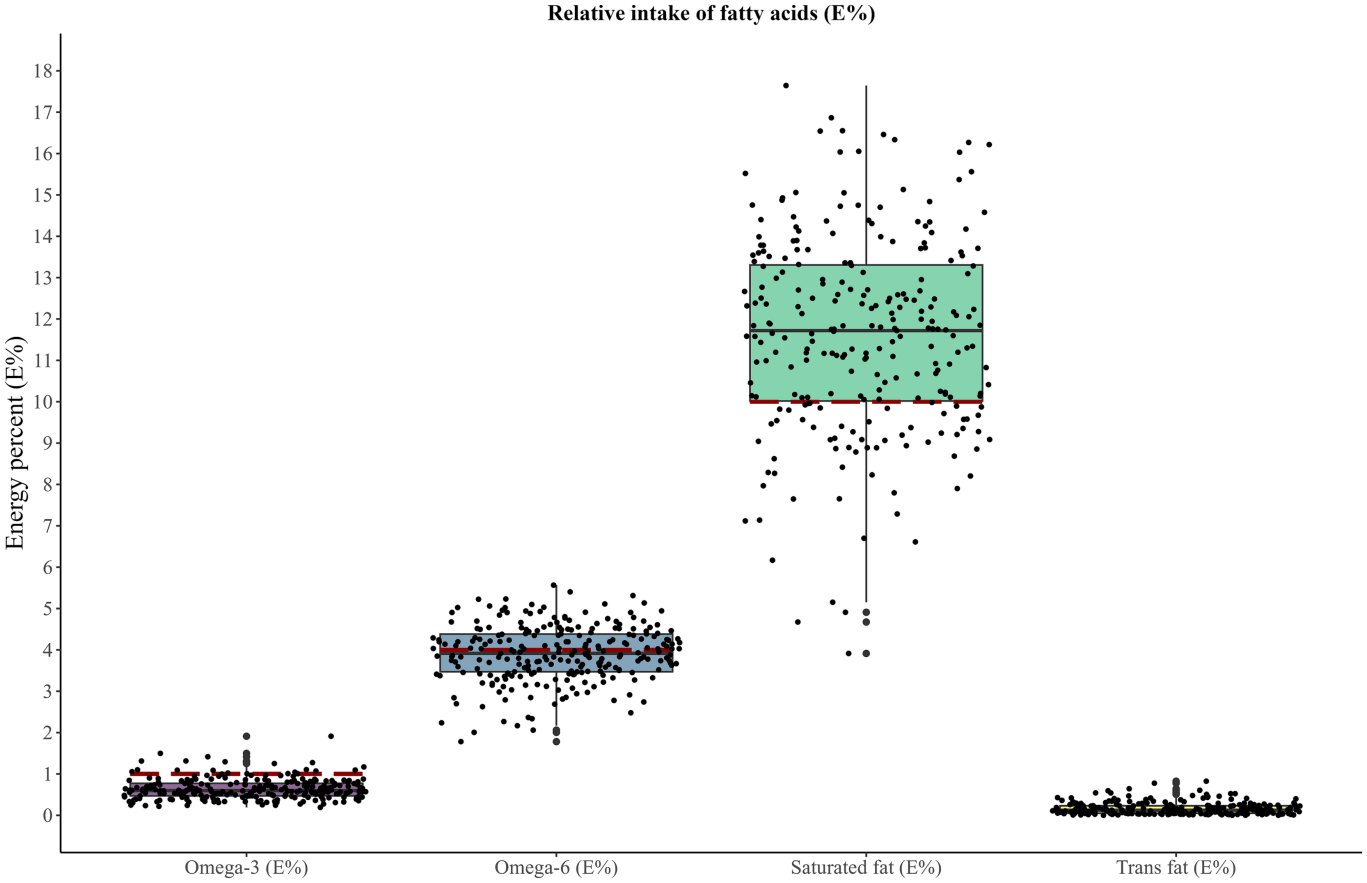
Reported intake of fatty acids in relation to recommended intakes from NNR 2023. The dashed horizontal lines represent the recommended intakes: Omega-3, ≥1E%; Omega-6, ≥4E%; Saturated fat, <10E. The recommendation for saturated fat is taken for children from 1 year of age and must be interpreted with caution. trans fat is recommended to be kept as low as possible, hence, no horizontal line.

Regarding specific fatty acids, the recommended intake for this age group is that *n*-3 fatty acids should contribute to at least 1E%, *n*-6 fatty acids to at least 4E% and trans fat should be kept as low as possible. A recommendation regarding saturated fat is not set for children of this age, but from 1 year of age, it is advised to keep the intake below 10E%. The children had a lower than recommended intake of omega-3 fatty acids [median (25th–75th percentile): 0.60 (0.47–0.77) E%], and omega-6 fatty acids [median (25th–75th percentile): 3.92 (3.47–4.38) E%], but a higher than recommended intake of saturated fatty acids [median (25th–75th percentile): 11.7 (10.0–13.3) E%] (Figure 2). The number of children fulfilling the recommended intakes of omega-3, omega-6, and saturated fat were 22 (9%), 116 (46%), and 63 (25%), respectively. To note, from 1 year of age, the recommended intake of omega-3 changes from 1E% to 0.5E% and applying the latter limit would mean that 117 (47%) of the children had an adequate intake.

#### 3.3.2 Micronutrient intake in relation to recommendations

**Figure 3:**
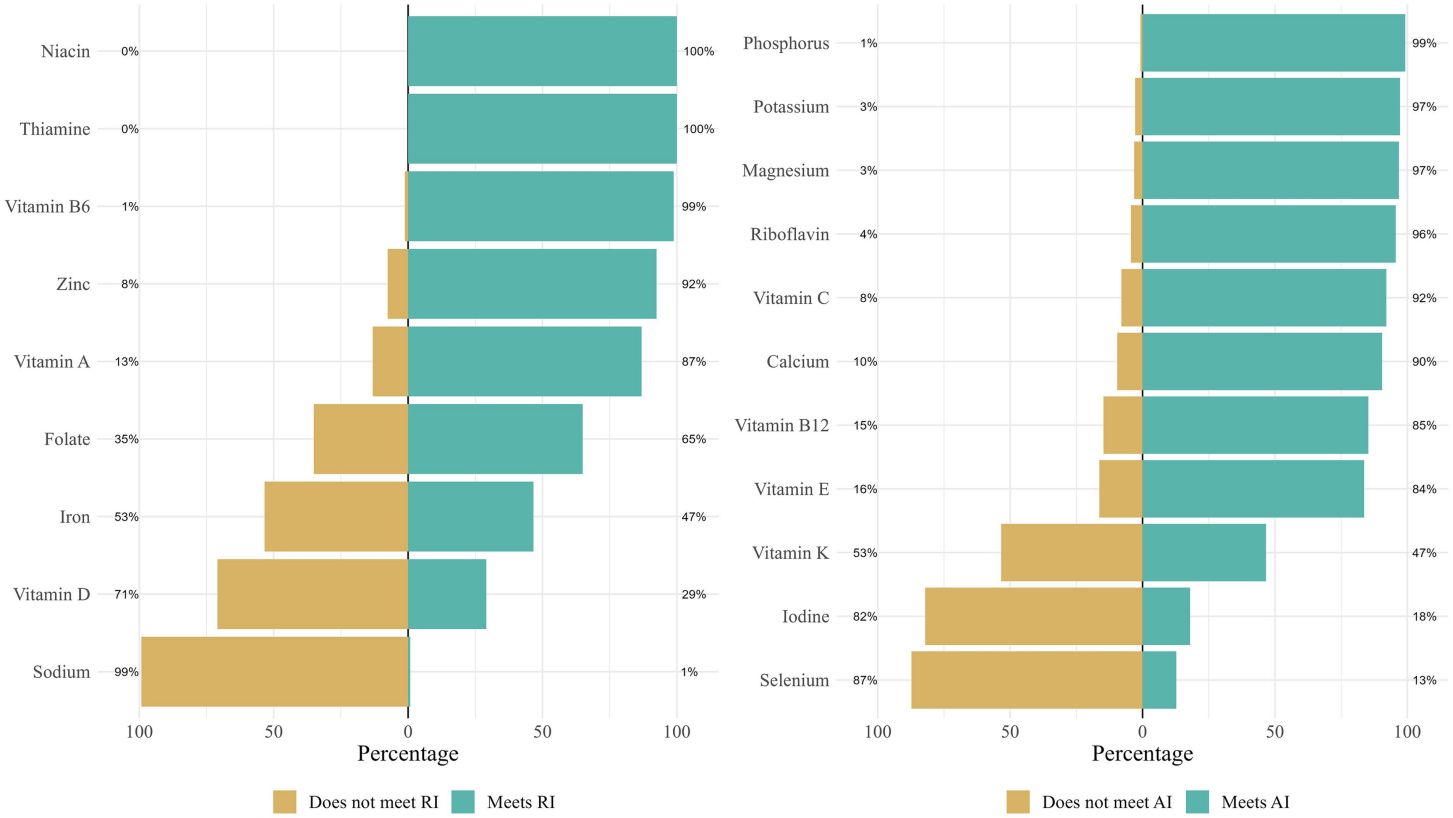
Reported intake of micronutrients from dietary sources in relation to recommended intakes from NNR 2023. The left plot shows how many of the 251 children who met the recommended intakes (RI). The right plot depicts adequate intakes (AI) since RI cannot be estimated due to limited evidence. Children breastfed or formula-fed at 10 months of age or later were excluded. The plot was created using the *likert* package in R. The following cut-offs were used: Niacin, ≥1.6 NE/MJ; Thiamine, ≥0.1 mg/MJ; Vitamin B6, ≥0.4 mg; Zinc, ≥3.0 mg; Vitamin A, ≥250 μg (RI given as 250 retinol equivalents, interpret with caution); Folate, ≥90 μg; Iron, ≥10 mg; Vitamin D, ≥10 μg; Sodium, ≤370 mg; Phosphorus, ≥170 mg; Potassium, ≥700 mg; Magnesium, ≥80 mg; Riboflavin, ≥0.4 mg; Vitamin C, ≥30 mg; Calcium, ≥310 mg; Vitamin B12, ≥1.5 μg; Vitamin E, ≥5 mg (AI is given in alpha tocopherol equivalents, interpret with caution); Vitamin K, ≥10 μg; Iodine, ≥85 μg (AI is given as a range 80–90 μg, middle value chosen in here); Selenium, ≥20 μg.

For some micronutrients, the Nordic Nutrition Recommendations provide recommended intake (RI) levels, while for others with scarce evidence, an adequate intake (AI) is provided. The number of children reaching the RI (or AI in the absence of a RI) is presented in Figure 3 for the 251 children who were no longer breastfed or formula-fed at 10 months of age.

The majority of children did not meet the recommended intake levels for selenium (87%), iodine (80%), vitamin D (71%), iron (53%), and vitamin K (53%). However, most children reached the recommended levels for B vitamins (e.g., thiamine, riboflavin, niacin, vitamin B6, and B12), as well as for other essential nutrients such as potassium, phosphorus, magnesium, zinc, vitamin C, calcium, vitamin A, and vitamin E (Figure 3).

Almost all children had a higher sodium intake than recommended. When compared with the recommended maximum intake for children at this age (up to 11 months), only two children had a daily intake lower than 370 mg (Figure 3). To note, when applying the recommendation given for older children (1–3 years, 1,100 mg), 117 (47%) children had a low enough intake to reduce the risk of chronic diseases.

#### 3.3.3 Dietary supplements

The food frequency questionnaire included a general question regarding the child’s use of vitamins, minerals, or other supplements. If any consumption was reported, the parents were asked to specify what type of supplement the child consumed (e.g., multivitamins, iron supplements, or folic acid) and how often. Supplement intake was not included in the nutritional calculations since the dose and type might vary largely even within the same group of supplements.

**Figure 4:**
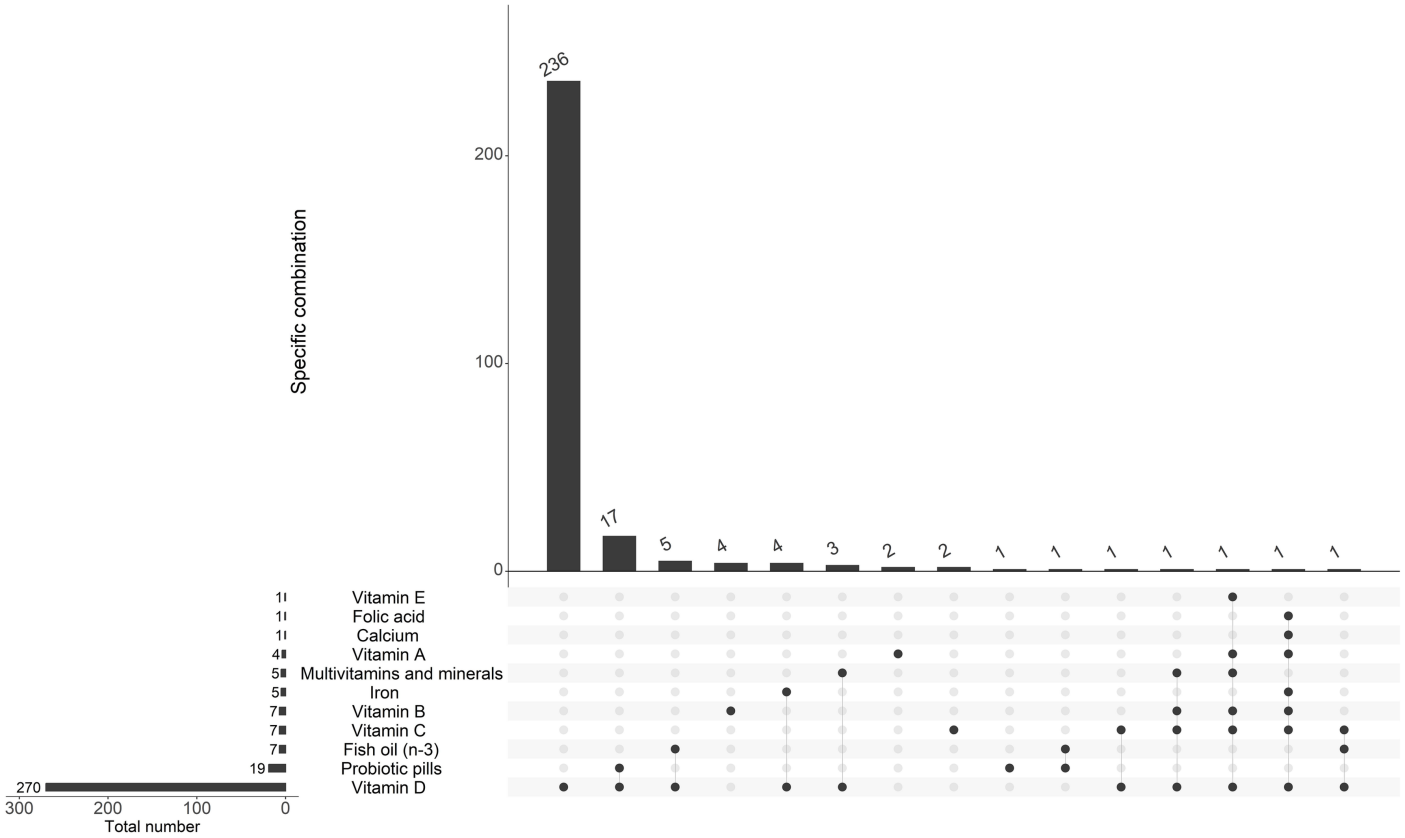
Supplement use among the 280 children receiving any supplements. The main bar chart displays the number of children who received a specific combination of supplements as indicated below the chart with black dots. The bar chart in the bottom left specifies the total number of children receiving the supplement written on the same line to the right, regardless of other combinations.

In Figure 4, the use of supplements at 1 year of age is visualized. Out of the 523 children included in this study, 243 (46%) had regular consumption, 37 (7%) sporadic consumption, 236 (45%) had no supplement consumption, and data was missing for seven children. Regarding the children who did not receive any breast milk or formula, 117 (47%) had regular consumption, 17 (7%) sporadic, and 114 (45%) had no supplement consumption, and information was missing for three of the children. Vitamin D (without additional vitamins or minerals) was the far most common supplement given to children (236 out of all 280 supplement users, 84%, Figure 4; and 119 out of the 134 supplement users who did not receive breast milk or formula at 10 months or later, 89%, Supplementary Figure S2). If supplement use was reported, most children consumed it daily. For instance, among the 270 children who reported any vitamin D supplements, 76% consumed it daily, followed by 21% who consumed it a few times a week. The only supplement not consumed daily by the reported users was fish oil (omega-3), which 43% consumed a few times a week.

### 3.4 Family characteristics and food and nutrition intake

**Figure 5:**
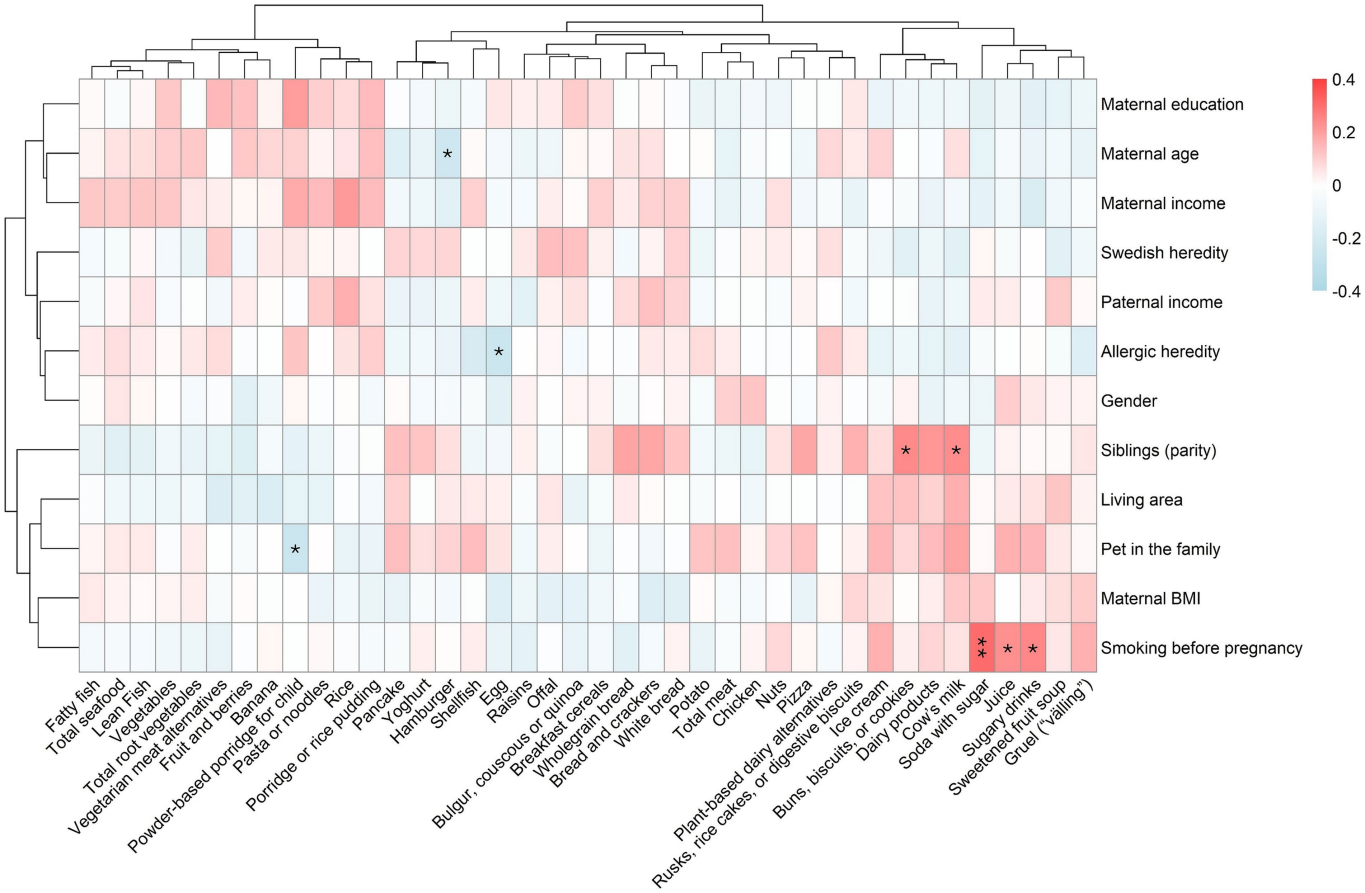
Spearman correlation between family characteristics and food intake collected with a food frequency questionnaire sent out at 1 year of age. Includes 251 children who were not fed breast milk or formula after 10 months of age (i.e., diet as the only source of energy). All *p*-values were adjusted for multiple testing using the FDR method. Significant correlations are denoted with asterisks as follows: *p* < 0.001 = ***, *p* < 0.01 = ** and *p* < 0.05 = *.

**Figure 6:**
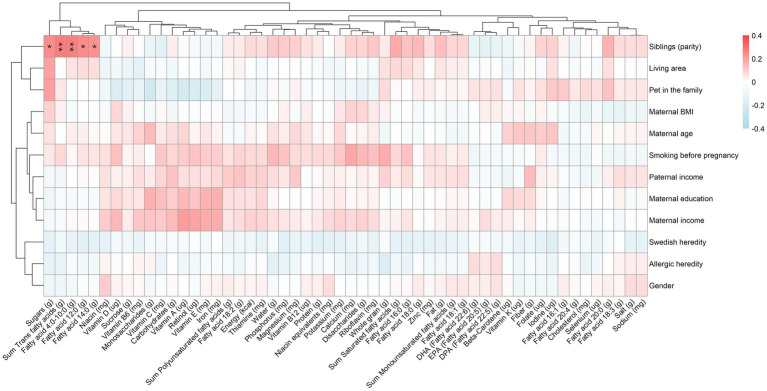
Spearman correlation between family characteristics and intake of nutrients calculated based on the whole diet at 1 year of age. Adjusted for multiple testing using the FDR method. Includes 251 children who were not fed breast milk or formula after 10 months of age (i.e., diet as the only source of energy). Significant correlations are denoted with asterisks as follows: *p* < 0.001 = ***, *p* < 0.01 = ** and *p* < 0.05 = *.

To investigate if family characteristics are related to what children eat at the end of their first year, Spearman correlation analyses were conducted between family characteristics and food intakes. Complementary Mann–Whitney *U* or Kruskal–Wallis tests were conducted for categorical variables which were found significant in the correlation analyses, and the results are presented in text together with the median intakes of the different groups. The results from the correlation analyses are summarized in Figure 5, which shows a reduced number of food items for easier visualization. All statistically significant associations are included in the figure. All results (i.e., more food items) are presented fully in detail in Supplementary File 1. Corresponding results, but for the nutrient intakes calculated based on the whole diet, are presented in Figure 6 and Supplementary File 2.

#### 3.4.1 Factors associated with food intake

Some family characteristics seemed to be associated with the children’s food intake (Figure 5 and Supplementary Data). Correlation coefficients were used to visualize the directionality of consumptions, and significant results after adjustment for multiple testing are presented in the text together with median (25th–75th percentile) and unadjusted *p*-value from complementary Mann–Whitney *U* (binary variables) or Kruskal–Wallis (multilevel variables) analyses.

Having a mother who smoked before the pregnancy was associated with higher consumption of sugary drinks [0 (0–0) grams/day versus 0 (0–39) grams/day, *p* < 0.001]. To note, in the group of children to non-smoking women (*N* = 229) 11% had any consumption of sugary drinks compared to 40% of the children to smoking mothers (*N* = 20). None of the children of non-smoking women consumed any soda, and 7% consumed juice, which can be compared to the children of the smoking mothers, where two consumed sugar-sweetened soda, one soda sweetened with artificial sweeteners, and 30% consumed juice.

Further, having pets in the family was associated with a lower consumption of powder-based porridge [0 (0–130) grams/day versus 130 (0–130) grams/day, *p* < 0.001], and having any allergy within the family was associated with a lower consumption of egg [4 (0–11) grams/day versus 11 (4–11) grams/day, *p* < 0.001]. Among the 160 children with allergic heredity, 57% consumed eggs, while 83% of the 80 children without allergic heredity had such consumption. Among the children with any older siblings (*N* = 127), 47% had any consumption of buns, biscuits, or cookies, and 58% any intake of cow’s milk, while corresponding numbers for children without siblings (*N* = 123) were 26 and 38%, respectively. Also, a higher age of the mother was related to lower consumption of hamburgers.

No associations were found between food intake at 1 year and living area, maternal education, BMI, nationality, income, paternal income, or gender of the child. All associations, including non-adjusted associations, are presented in detail in Supplementary Data.

#### 3.4.2 Factors associated with nutrient intake

In Figure 6 associations between family characteristics and the children’s nutrient intake are visualized. As can be seen, the only factor associated with any of the nutrients was the presence of older siblings which was positively related to the intake of trans fats, sugars, and several different saturated fatty acids.

More specifically, having older siblings was associated with a higher intake of trans fatty acids [0.15 (0.06–0.26) grams/day versus 0.07 (0.03–0.16) grams/day, *p* < 0.001], sugars [0.47 (0.00–4.68) grams/day versus 0.00 (0.00–0.54) grams/day, *p* < 0.001], and the saturated fatty acids C4:0–10:0 [0.58 (0.28–0.95) grams/day versus 0.31 (0.15–0.67) grams/day, *p* < 0.001], C12:0 (i.e., lauric acid) [0.34 (0.22–0.46) grams/day versus 0.26 (0.18–0.36) grams/day, *p* < 0.001], and C14:0 (i.e., myristic acid) [0.98 (0.60–1.33) grams/day versus 0.66 (0.50–1.10) grams/day, *p* < 0.001] (Figure 6). No other family characteristics were significantly associated with the nutrient intake at 1 year (Figure 6 and Supplementary Data).

## 4 Discussion

This study examined the dietary intake of one-year-old children participating in the NICE prospective birth cohort. We also assessed how these intakes align with the Nordic Nutrition Recommendations and relate to family characteristics. Our findings revealed a significant shortage in dietary intake of several essential micronutrients. Specifically, 87% of children did not meet the recommendations for selenium, 80% for iodine, 71% for vitamin D, 53% for iron, and 53% for vitamin K, however, a majority of the children received vitamin D supplements. The low intakes of selenium, iodine, and iron are especially concerning given their critical roles in child development. Selenium, iodine, and iron are all essential for thyroid function (23), and deficiencies in these trace elements can lead to severe consequences including impaired cognitive development and stunted growth (10, 11, 23, 24).

When putting the micronutrient intakes into perspective, our findings reveal some notable differences compared to the Finnish VIDI study of non-breastfed one-year-olds (25). Despite a slightly lower energy intake in our study (814 kcal versus their 881 kcal), we report 39% higher iron intake (9.6 mg versus their 6.9 mg) and 11% higher vitamin D intake (8.3 μg versus their 7.5 μg). On the other hand, the children in our study had 30% lower zinc intake (4.5 mg versus their 6.4 mg). It is important to note that the Finnish study used three-day food records, and the children were enrolled in a vitamin D intervention conducted shortly after birth. These differences in study designs might limit accurate comparisons with our results, which were generated using a food frequency questionnaire with children enrolled in a prospective birth cohort. Unlike the three-day food records that provide a detailed snapshot of dietary intake on specific days, the food frequency questionnaire captures dietary intake over a longer period. Additionally, the Finnish study did not report data on micronutrients of potential interest, such as iodine and selenium. Interestingly, when comparing with the Swedish Food Agency’s latest investigation of 1.5-year-olds called Riksmaten (*N* = 1,078) (7), the reported intakes of iron, specifically, in our study is notably higher (9.6 mg versus their 6.7 mg), perhaps due to the higher consumption of iron fortified porridge at an earlier age. In a subgroup of children in their survey (*N* = 249), they also measured plasma ferritin and found that 6% had levels indicating iron deficiency. Despite this, they conclude that the risk of iron deficiency among Swedish children is low (7).

The low intake of selenium, iodine, iron, and vitamin D in this cohort may be explained by the dietary patterns observed among the children. Seafood, a good source of these micronutrients, was consumed at an estimated 20 g per day. To meet the recommended intakes of selenium and iodine, seafood consumption would need to increase to 45 and 25 g per day, respectively, using boiled cod with salt as a proxy (17). However, at such a low age when the overall intake of food is relatively low, meeting nutritional needs simply by increasing the intake of one food item is not realistic, and choosing nutrient-dense food is therefore of extra importance. For instance, choosing black pudding over minced meat now and then would quickly help meet the iron needs of the child with an intake of around 50 g being enough to fulfill the estimated need (17). Also, choosing home-made oatmeal with rolled oats, water, and iodized salt instead of ready-to-eat child porridges would require half the amount in order to meet the iodine need (210 g versus 440 g), while this switch would make iron needs impossible to meet simply with porridge due to the lack of iron fortification (0.6 mg iron per 100 g oatmeal, compared to 1.7 mg in the fortified ready-to-eat porridge) (17).

The sodium intake in this group of children probably comes from non-fortified sources, considering that the sodium intake was higher in our cohort than recommended despite the low iodine intake. The primary source of iodine in this area is iodized salt but the recommendation for children under 1 year of age is to avoid adding salt to their food (26). Notably, 6 months later, at 18 months of age, the parents reported the type of salt used at home. A significant majority (88%) of the families used iodized salt, and 33% of the children received salt in their food but to a lower extent than the rest of the family, while 8% still avoided added salt. Despite the importance of consuming iodized salt to ensure adequate iodine intake, salt is the main source of sodium, which was higher than recommended in our study. Therefore, in order to meet the iodine needs without exceeding the sodium intake, other sources of iodine such as lean fish, dairy products, and eggs might be suitable choices.

Regarding the findings that the children may not receive adequate amounts of Vitamin K, it is important to note that our nutritional calculations rely on data from the Swedish Food Agency’s database (17). In Riksmaten, the agency excluded Vitamin K from their report with the motivation that not all food items in their database have information on their Vitamin K content (7). Therefore, the actual intake of Vitamin K in our cohort could be higher than estimated, since these rely on the same database. Additionally, all newborns in Sweden receive an intramuscular injection of Vitamin K, indicating a low risk of deficiency. Thus, our results regarding Vitamin K should be interpreted with caution.

Fatty acid intake was found to be suboptimal in this cohort, with median intakes of omega-3 fatty acids (0.60 E%) and omega-6 fatty acids (3.92 E%) falling below the recommended levels. These low intakes are concerning as essential fatty acids play a critical role in brain development, particularly during the fetal stage and the first year of life (27), when enhanced accumulation of fatty acids in the brain occurs (28). Proper neurodevelopment during this period is dependent on adequate nutrition, with essential fatty acids being vital for membrane structure and cell signaling (29). Deficiency in omega-3 fatty acids has in clinical trials been clearly linked to, e.g., worse symptoms among children with attention deficit hyperactivity disorder (ADHD) (30). The lower fat intake observed in our study (29 E% versus the recommended 30–45 E%) aligns with findings from non-breastfed one-year-olds in the Finnish DIPP study (31). Although the clinical significance of such a small deviation in fat intake is not fully understood, it is important to monitor potential effects on neurodevelopment.

In addition to the low-fat intake, protein intake was higher than recommended, contributing 17% of total energy intake compared to the recommended 7–15%. High protein intake has been associated with an increased risk of childhood obesity. A meta-analysis estimated that an increment of 1 E% protein could increase BMI by approximately 0.06 kg/m^2^ (32). Based on our findings, this would translate to a potential BMI increment of 0.12–0.60 kg/m^2^ in these children. The combined pattern of lower fat and higher protein intake underscores the need to inform families about nutritional guidelines, emphasizing the risks associated with both restricted and excessive nutrient intakes.

The Swedish Food Agency’s dietary survey of 1.5-year-old children provide a valuable reference point for our findings (7). Despite the half-year age difference, this survey is particularly relevant given the scarcity of comparative studies on one-year-olds in Sweden. The 1.5-year-old children were taller (81 cm vs. 76 cm), heavier (11.6 kg vs. 10.1 kg), and had a higher overall energy intake (1,171 kcal vs. 832 kcal) than the children in our cohort, making direct comparisons with our findings challenging in terms of absolute values. Interestingly, both studies observed a similar trend in energy intake between boys and girls, with boys consuming approximately 6% more energy than girls, compared to the 4% difference identified in our study. Regarding macronutrient distribution, their survey reported higher relative intakes of fat (36E% versus 29E%), but lower intakes of protein (15E% versus 17E%) and carbohydrates (47E% versus 51%) than observed in our study.

The secondary aim of this study was to investigate the relationship between family characteristics and the children’s food intake, identifying potential lifestyle factors for targeted interventions and future research. We observed significant associations between food intake at the end of the first year and two key family characteristics: the presence of older siblings and maternal tobacco smoking before pregnancy. We have previously reported that these two factors seemed related to what the mothers ate themselves during pregnancy, with a similar correlation pattern between parity and a higher intake of cow’s milk, and smoking with a higher intake of sugary drinks (15). Previous studies have shown that maternal food habits influence what their children eat (33, 34), and since we saw similar patterns for the mothers themselves (15), it is perhaps not so surprising that they also relate to the children’s food intake. Another recent study investigated the association between parental smoking and children’s food intakes and saw a clear relationship between more unhealthy food choices, although the children of that study were much older than in ours (35).

Importantly, when comparing the responders (i.e., included) with the non-responders (i.e., excluded), the ones included in our analyses had higher educated mothers (72% versus 54% with university degree, *p* = 0.007) and seemed less likely to have smoking mothers, although not statistically significant (95% versus 88%, *p* = 0.071). Further, the included mothers were slightly older than the non-responders [median (25th–75th percentiles): 30 (27–34) versus 29 (26–32) years, *p* = 0.049]. Hence, the differences between the included families in this study are similar to the differences as previously reported for the whole cohort (36).

### 4.1 Strengths and limitations

Our study has several notable strengths, including a comprehensive collection of dietary data and family characteristics. The dietary information was meticulously quantified using both intake frequency and portion size representations. Additionally, the high response rate of the food frequency questionnaire enabled us to concentrate exclusively on non-breastfed and non-formula-fed children. We also adjusted for multiple testing, thereby enhancing the validity of our findings.

However, we must acknowledge the inherent challenges in measuring diet. Dietary intake data were collected using a non-validated food frequency questionnaire, which may be subject to recall bias and might not accurately reflect actual intake in this specific population. The estimation of what was served to the child might not accurately reflect what was actually consumed. Furthermore, the generalizability of our findings may be constrained, given that all participating children were from Norrbotten County, with a specific focus on those not receiving breastmilk or formula by the end of their first year. Nevertheless, we included results for all children, irrespective of weaning practices, to facilitate comparability with future studies. Also, regarding generalizability, there seems to be a self-selection bias in this study as we have previously reported, with more highly educated families responding to the questionnaires (36).

## 5 Conclusion

Children in Northern Sweden consume a diverse range of foods by the end of their first year, however, their dietary quality needs improvements. Intakes of critical micronutrients such as selenium, iodine, and iron were substantially below recommended levels, posing potential risks to growth and neurodevelopment. Additionally, the dietary patterns observed, characterized by higher protein and lower fat intake compared to recommendations, highlight the need for nutritional guidance to mitigate long-term health risks, such as childhood obesity and suboptimal brain development. Moreover, family dynamics, including the presence of siblings and maternal smoking habits, should be considered in designing effective dietary interventions, as these factors may be indicative of the context in which feeding practices are established.

## Data Availability

The datasets presented in this article are not readily available because they relate to information that could compromise research participant privacy or consent. Explicit consent to deposit raw data was not obtained from the participants. Therefore, the data can only be made public if a new consent is filled in by the participants together with a new ethical permit being obtained. The R-codes for conducting the statistical analyses can be obtained from: https://gitlab.com/miastravik/. Requests to access the datasets should be directed to Ann-Sofie Sandberg, ann-sofie.sandberg@chalmers.se.
