# Supplementary material for: Food and nutrient intake at 1 year of age in Northern Sweden: results from the Swedish NICE birth cohort
Source: Front Nutr. 2025 Feb 13;12:1548512. doi: 10.3389/fnut.2025.1548512 (PMC11864951; doi:10.3389/fnut.2025.1548512)
Supplement: Supplementary file 1 [file Data_Sheet_1.docx]

Supplementary Material

Food and Nutrient Intake at 1 year of age in Northern Sweden: Results from the Swedish NICE birth cohort

Mia Stråvik^1*^, Mariza Kampouri^2^, Klara Gustin^2^, Anna Sandin^3^, Agnes E. Wold^4^, Malin Barman^1^, Ann-Sofie Sandberg^1^

^1^Department of Life Sciences, Food and Nutrition Science, Chalmers University of Technology, Gothenburg, Sweden

^2^Institute of Environmental Medicine, Karolinska Institutet, Stockholm, Sweden

^3^Department of Clinical Science, Pediatrics, Sunderby Research Unit, Umeå University, Umeå, Sweden

^4^Department of Infectious Diseases, Institute of Biomedicine, Sahlgrenska Academy, University of Gothenburg, Gothenburg, Sweden

# Supplementary Data

**Excel file** **1**. Correlation Matrix with all food items and family characteristics: Spearman correlation with correlation coefficients, p-value and adjusted p-value.

**Excel file** **2**. Correlation Matrix with all nutrients and family characteristics: Spearman correlation with correlation coefficients, p-value and adjusted p-value.

# Supplementary Tables and Figures

## Tables

**Supplementary Table 1**. Background to quantification (grams/day) and creation of food groups.

**Supplementary Table 2**. Energy needs and intakes of all children in the NICE cohort (N=523).

**Supplementary Table 3**. Food intake (grams/day) of all 1-year-old children in the NICE cohort.

**Supplementary Table 4.** Micro- and macronutrient intakes (per day) of all children in the NICE cohort (N=523).

## Figures

**Supplementary Figure 1.** Breastfeeding and formula use leading to exclusion from primary analyses.

**Supplementary Figure 2.** Supplement use among the 251 children who did not receive breast milk or formula.

**Supplementary Table 1**. Background to quantification (grams/day) and creation of food groups.

| **Food item** | **Question** | **Assumptions for quantification** | **Additional information** |
| --- | --- | --- | --- |
| **Candy** | Chocolate, candy, or natural candy | Background for calculations   - 1 bar of “Kinder Maxi” = 21 grams (producer) - 1 box of “Zoo Tablettask” or “Fruxo Tablettask” = 20 grams - 1 “Kinder Surprise Ägg” = 20 grams   Weighted for estimations^1^   - 1 Marabou square = 6 grams   Assumption   - “Enstaka godisbitar eller chokladrutor” ≈ 20 grams | Question regarding intake frequency was followed by a question of how much the child consumes at each intake occasion.  The lowest alternative was ”Enstaka godisbitar eller chokladrutor” which warrants an assumption regarding what “enstaka” is. |
| **Chips** | Chips, popcorn, or cheese doodles | Weighted for estimations^1^   - 6 Chips (Gårdschips) = 8 grams   Assumption:   - “Enstaka chips, popcorn eller ostbågar” = 8 grams | Question regarding intake frequency was followed by a question of how much the child consumes at each intake occasion.  The lowest alternative was ”Enstaka chips, popcorn eller ostbågar” which warrants an assumption regarding what “enstaka” is |
| **Ice cream** | Ice cream | Weighted for estimations^1^   - 1 Small-to-medium scope = 33 grams - 1 Large scope = 55 grams   Assumptions:   - “1 kula” = 35 grams - “Mindre än 1 kula” = ½ kula | Question regarding intake frequency was followed by a question of how much the child consumes at each intake occasion.  The alternatives were formulated as number of scopes which warrants an assumption regarding the size of “1 scope” |
| **Probiotics** | Products with probiotics | Background for calculations   - 1 “Actimel Drickyoghurt Jordgubb” = 100 grams - 1 portion “Filmjölk A-fil fett 3% berikad” = 200 grams (DietistNet) - 1 medium-sized portion “Fruit Drink, Proviva, Mango, Contains Probiotics” = 200 grams (Fineli) - *Mean: 167 grams*   Assumptions:  1 portion ≈ 165 grams |  |
| **Sandwich toppings** | Sandwich toppings such as cheese, ham | Assumptions:   - The subtypes (except for cucumber, tomato, and “other”) are consumed equally frequently if several bread toppings were reported - Cucumber and tomato are handled as complementary (used in combination with other toppings) and not unique toppings - “Other” topping is not included in the calculations due to its ambiguous nature | We have asked about how often they have *something* on the bread. If they reported any intake, they got follow-up questions regarding different toppings in yes/no format.  Those without any follow-up responses on bread topping are coded as SYSMIS instead of 0, so all with zero have replied that they use topping but not that specific kind. |
| Cheese | Cheese | Background for calculation   - 1 slice (2 mm) "Ost hårdost fett 31%" = 10 grams (DietistNet) - 1 portion "Ost hårdost fett 31%" = 15 grams (DietistNet)   Assumptions:   - 1 portion = 1-2 slices ≈ 15 grams |  |
| Ham | Deli meat (e.g., ham or salami) on bread | Background for calculation   - 1 slice "Gris skinka rökt fett 6%" = 15 grams (DietistNet)   Assumptions:   - 1 portion = 1 slice |  |
| Marmalade | Marmalade | Background for calculation   - 1 tbsp “Apelsinmarmelad” ≈ 18 grams (DietistNet)   Assumptions:   - 1 portion = 1 tbsp |  |
| Liver pâte | Liver pâté | Background for calculation   - 1 tbsp “Leverpastej bredbar fett ca 10%” = 13 grams (DietistNet) - 1 portion “Leverpastej bredbar fett ca 10%” = 15 grams (DietistNet)   Assumptions:   - 1 portion = 15 grams |  |
| Caviar | Caviar (cod roe spread) | Background for calculation   - 1 tbsp “Påläggskaviar original” = 15 grams (DietistNet) - 1 portion “Påläggskaviar original” = 10 grams (DietistNet)   Assumptions:   - 1 portion = 10 grams |  |
| Messmör (whey cheese) | Messmör (brown whey cheese spread) | Background for calculation   - 18 grams/tbsp “Messmör fett 5% berikad” (DietistNet) - 18 grams = 1 portion “Messmör fett 5% berikad” (DietistNet)   Assumptions:   - 1 portion = 18 grams |  |
| Tomato | Tomato or bell pepper | Background for calculation   - 80 grams/tomato “Tomat” (DietistNet)   Weighted for estimations^1^   - 1 small tomato (smaller than the great majority in the store, weighed around 50 tomatoes in store >80 grams before finding one matching the DietistNet standard) = 80 grams - The middle slice (i.e., the only slice looking like a full piece) after cutting it in even pieces = 20 grams - The weight vary greatly between pieces since the diameter varies (min-max: 6-20 grams)   Assumptions:   - Tomato and cucumbers are complementary items when reported as toppings to bread (i.e., not used *instead* of other toppings). - Tomato and cucumbers are used every other time - 1 slice of tomato = 1 portion - It is difficult to buy a tomato weighing less than 100 grams - 1 slice ≈ 20 grams |  |
| Cucumber | Cucumber | Background for calculation   - 1 slice “Gurka” = 5 grams (DietistNet)   Weighted for estimations^1^   - 1 slice (4 mm) of cucumber ≈ 5 grams   Assumptions:   - Tomato and cucumbers are complementary items when reported as toppings to bread (i.e., not used *instead* of other toppings). - Tomato and cucumbers are used every other time - 1 portion = 3 slices |  |
| **Bulgur, couscous or quinoa** | Bulgur, couscous, or quinoa |  | Picture of five different portion sizes of pasta. |
| **Rice** | Rice |  | Picture of five different portion sizes of pasta. |
| **Pasta or noodles** | Pasta or noodles |  | Picture of five different portion sizes of pasta. |
| **Total potato** | *Created food group*   - French fries or fried potato - Boiled or mashed potato |  |  |
| French fries or fried potato | Fries, potato patties, fried potatoes, potato gratin, or Swedish hash ("Pyttipanna") |  | Picture of five different portion sizes of pasta. |
| Boiled or mashed potato | Boiled potatoes or mashed potatoes |  | Picture of five different portion sizes of pasta. |
| **Vegetarian meat alternatives** | Vegetarian dishes e.g., lentil stew, bean patty, soy sausage or quorn | Background for calculation   - This is the general question (before follow-up regarding types) - Handled as a *total intake* variable for meat substitutes (asked within the meat questions) | Picture of five different portion sizes of meatballs. |
| Beans or lentils | Bean, lentil or chickpea dishes e.g., lentil stew, bean patty or falafel | Assumptions   - The subtypes are consumed equally frequently if several vegetarian meat alternatives were reported |  |
| Soy products | Soy meat, soy sausage or tofu | Assumptions   - The subtypes are consumed equally frequently if several vegetarian meat alternatives were reported |  |
| Quorn | Quorn mince or Quorn stew | Assumptions   - The subtypes are consumed equally frequently if several vegetarian meat alternatives were reported |  |
| Roots | Root vegetable dishes with e.g., beets, parsnips or Swedish turnip | Background for calculation   - This is a follow-up question to "Vegetariska rätter t.ex. linsgryta, bönbiff, sojakorv eller quorn" - Handled as a meat substitute since only those responding "yes" to the general *meat substitute* question get this follow-up this variable is not believed to capture an alternative to e.g., potato or vegetables, and therefore calculated as "kött, fisk eller motsvarande vegetariska alternativ".   Assumptions:   - The subtypes are consumed equally frequently if several vegetarian meat alternatives were reported |  |
| **Total seafood** | *Created food group*   - Fish or shellfish - Caviar (cod roe spread) |  | Picture of five different portion sizes of meatballs. |
| Seafood (without caviar) | Fish or shellfish | Background for calculation   - This general question (before follow-up regarding types) can be handled as a "total fish and shellfish" intake variable - Caviar used on bread is not included in this section |  |
| Lean Fish | *Created food group*   - Fish fingers - Cod or pollock - Tuna |  |  |
| Fish fingers | Fish fingers or fish balls (boiled fish mince, starch, milk) | Assumptions:   - The subtypes are consumed equally frequently if several fish and shellfish were reported |  |
| Cod or pollock | Cod, pollock or other white fish (not fish fingers or fish balls) | Assumptions:   - The subtypes are consumed equally frequently if several fish and shellfish were reported |  |
| Tuna | Tuna | Assumptions:   - The subtypes are consumed equally frequently if several fish and shellfish were reported |  |
| Fatty fish | Salmon, sushi, mackerel or herring | Assumptions:   - The subtypes are consumed equally frequently if several fish and shellfish were reported |  |
| Shellfish | Shellfish e.g., shrimps or mussels | Assumptions:   - The subtypes are consumed equally frequently if several fish and shellfish were reported |  |
| Other fish | Other fish | Assumptions:   - The subtypes are consumed equally frequently if several fish and shellfish were reported |  |
| **Total vegetables** | Vegetables, raw or cooked | Background for calculation   - Handled as a total intake variable and calculated based on picture of portion size as chosen by the participant | Picture of five different portion sizes of meatballs. |
| Tomato or bell pepper | Tomato or bell pepper | Assumptions:   - The subtypes are consumed equally frequently if several vegetables were reported - Tomato intake reported as follow-up question to topping on bread is assumed to already be included in that variable (i.e., not summarized due to the risk of double-reporting). |  |
| Cucumber or lettuce | Cucumber, lettuce or zucchini | Assumptions:   - The subtypes are consumed equally frequently if several vegetables were reported |  |
| Carrot | Carrots | Assumptions:   - The subtypes are consumed equally frequently if several vegetables were reported |  |
| Onion | Onion | Assumptions:   - The subtypes are consumed equally frequently if several vegetables were reported |  |
| Corn | Corn | Assumptions:   - The subtypes are consumed equally frequently if several vegetables were reported |  |
| Avocado | Avocado | Assumptions:   - The subtypes are consumed equally frequently if several vegetables were reported |  |
| Cauliflower or cabbage | Cauliflower or cabbage e.g., pizza salad | Assumptions:   - The subtypes are consumed equally frequently if several vegetables were reported |  |
| Broccoli | Broccoli | Assumptions:   - The subtypes are consumed equally frequently if several vegetables were reported |  |
| Spinach | Spinach | Assumptions:   - The subtypes are consumed equally frequently if several vegetables were reported |  |
| Green peas | Green peas | Assumptions:   - The subtypes are consumed equally frequently if several vegetables were reported |  |
| Beans, lentils or chickpeas | Beans, lentils or chickpeas | Assumptions:   - The subtypes are consumed equally frequently if several vegetables were reported |  |
| Parsnip, Swedish turnip or celeriac | Parsnip, Swedish turnip or celeriac | Assumptions:   - The subtypes are consumed equally frequently if several vegetables were reported |  |
| Pickled vegetables | Pickled vegetables | Assumptions:   - The subtypes are consumed equally frequently if several vegetables were reported |  |
| Other vegetables | Other vegetables | Assumptions:   - The subtypes are consumed equally frequently if several vegetables were reported |  |
| **Water** | Water (from tap or bottle) | Background for calculation   - 1 glass “Vatten kranvatten” = 200 grams (DietistNet)   Assumptions:   - 1 glass = 200 grams |  |
| **Sugary drinks** | *Created food group*   - Total soda - Juice |  |  |
| Total soda | Soda or "saft" (fruit syrup + water) | Background for calculation   - 1 portion “Saft drickf.” = 200 grams (DietistNet)   Assumptions:   - 1 glass = 200 grams |  |
| Sugar | Soda or "saft" (fruit syrup + water) with sugar | Assumptions   - The subtypes are consumed equally frequently if several sodas were reported | Note that several individuals did not specify what type of soda the consumed in the follow-up question and are therefore only represented in the total soda variable. |
| Artificial sweeteners | Diet soda or light "saft" (fruit syrup + water) with sweetener | Assumptions   - The subtypes are consumed equally frequently if several sodas were reported | Note that several individuals did not specify what type of soda the consumed in the follow-up question and are therefore only represented in the total soda variable. |
| Juice | Juice | Assumptions:   - 1 glass = 200 grams |  |
| **Plant-based dairy alternatives** | Plant-based alternatives to milk, such as from oats, soy, coconut, sesame seeds | Assumptions   - 1 glass = 200 grams |  |
| Oat drink | Oat drink | Assumptions:   - The subtypes are consumed equally frequently if several drinks were reported |  |
| Soy drink | Soy drink | Assumptions   - The subtypes are consumed equally frequently if several drinks were reported |  |
| Sesame drink | Sesame drink | Assumptions   - The subtypes are consumed equally frequently if several drinks were reported |  |
| Coconut milk | Coconut milk | Assumptions   - The subtypes are consumed equally frequently if several drinks were reported |  |
| **Yoghurt** | "Filmjölk" (fermented sour milk), yoghurt, or drinking yoghurts | Background for calculation   - 1 portion “Fruktyoghurt fett 2.5%”, “Yoghurt naturell fett 3% berikad” or “Filmjölk fett 3% berikad” = 200 grams (DietistNet)   Assumptions:   - 1 portion yoghurt = 1 portion beverage (i.e., 200 grams) |  |
| Plain | Plain "filmjölk" (fermented sour milk) or yoghurt | Assumptions   - The subtypes are consumed equally frequently if several yoghurts were reported |  |
| Flavored | Fruit, berry or vanilla flavored "filmjölk" (fermented sour milk) or yoghurt | Assumptions   - The subtypes are consumed equally frequently if several yoghurts were reported |  |
| Other yoghurt | Other "filmjölk" (fermented sour milk) or yoghurt | Assumptions   - The subtypes are consumed equally frequently if several yoghurts were reported |  |
| **Soup** | Soup e.g., tomato soup, pea soup or Goulash | Assumptions:   - 1 portion soup = 1 portion beverage (i.e., 200 grams) |  |
| **Total gruel (“välling”)** | Gruel ("välling") | Background for calculation   - 1 portion = 2 dl water + 5 tbsp powder (product information for "Havrevälling mild 8m" and "Fullkornsvälling mild med havre" from Semper)   Weighted for estimations^1^   - 2 dl water + 5 tbsp powder = 228 grams ≈ 225 grams   Assumptions:   - The gruel is bought as powder from Semper - The gruel is prepared according to dosage recommendation on the packages |  |
| Mild whole grain | Mild whole grain gruel ("Mild fullkornsvälling") | Assumptions:   - The subtypes are consumed equally frequently if several gruels were reported |  |
| Whole grain | Whole grain gruel ("Fullkornsvälling") | Assumptions:   - The subtypes are consumed equally frequently if several gruels were reported |  |
| Corn gruel or gluten-free | Corn gruel or gluten-free gruel ("Majsvälling eller glutenfri välling") | Assumptions:   - The subtypes are consumed equally frequently if several gruels were reported |  |
| Other gruel | Other gruel ("Annan välling") | Assumptions:   - The subtypes are consumed equally frequently if several gruels were reported |  |
| **Porridge or rice pudding** | Porridge or rice pudding ("Gröt eller risifrutti") |  |  |
| Oatmeal | Oatmeal porridge ("Havregrynsgröt") | Background for calculation   - 1 portion “Havregrynsgröt fullkorn” = 200 grams (DietistNet)   Assumptions   - The subtypes are consumed equally frequently if several porridges were reported |  |
| Semolina or rice | Semolina porridge or rice porridge ("Mannagrynsgröt eller risgrynsgröt") | Background for calculation   - 1 portion “Risgrynsgröt” = 225 grams (DietistNet)   Assumptions   - The subtypes are consumed equally frequently if several porridges were reported |  |
| Snack e.g., rice pudding | Snack porridge e.g., Risifrutti (rice pudding) ("Mellanmålsgröt t.ex. risifrutti") | Background for calculation   - 1 “Risifrutti Original Jordgubb” = 175 grams (producer)   Assumptions   - The subtypes are consumed equally frequently if several porridges were reported |  |
| Powder-based for child | Powder-based porridge for children e.g., whole grain porridge or fruit porridge ("Barngröt pulverbaserad t.ex. fullkornsgröt eller fruktgröt") | Background for calculation   - 1 portion “Havregröt med päron & banan 8m” and “Fullkornsgröt mild & naturell 8m” = 1 dl water + 30 grams powder (producer) ≈ 130 grams   Assumptions   - The subtypes are consumed equally frequently if several porridges were reported |  |
| Other porridge | Other porridge ("Annan gröt") | Assumptions   - Unspecified porridge (“Annan gröt”) weighs the mean of all other subtypes = 182.5 grams - The subtypes are consumed equally frequently if several porridges were reported |  |
| **Nuts** | Nuts | Background for calculation   - Swedish Food Agency recommend avoidance of whole nuts and state that it should be in the form of e.g., peanut butter - 1 portion “Jordnötssmör” = 15 grams (DietistNet)   Weighted for estimations^1^   - 1 almond = 1 gram   Assumptions   - 1 portion = 1 tbsp nut butter ≈ 15 grams |  |
| **Buns, biscuits, or cookies** | Buns, biscuits, or cookies | Background for calculation   - 1 "Sött vetebröd m. fyllning bulle längd" = 25 grams (DietistNet) - 2 "Mariekex" = 10 grams (DietistNet) - 1 "Småkakor olika sorter" = 10 grams (DietistNet) - 1 "Giffel" = 22 grams (producer) - *Mean: 17 grams*   Assumptions   - 1 portion = 17 grams |  |
| **Raisins** | Raisins | Background for calculation   - The most commonly bought snack packs of raisins are "Russin Kärnfria 6-p ICA Russin” and “Sun Maid” - 1 box "Russin Kärnfria 6-p ICA Russin” = 42 grams - 1 box “Sun Maid” = 42.5 grams   Assumptions   - Sales from ICA Kvantum Stormarknad Luleå is representative for the study population - A child is given a box to consume at their own pace, and the portion size is therefore based on the size of the boxes |  |
| **Egg** | Egg | Background for calculation   - 1 boiled egg = 50 grams (DietistNet) - 1 raw egg = 55 grams (DietistNet)   Assumptions   - Egg is consumed as boiled - 1 portion = 1 egg = 50 grams |  |
| **Bread and crackers** | *Created food group*   - Total bread - Rusks, rice cakes, or digestive biscuits |  |  |
| Total bread | *Created food group*   - Total wholegrain bread - White bread |  |  |
| Total wholegrain bread | *Created food group*   - Soft wholegrain bread - Crispbread |  |  |
| Soft wholegrain bread | Whole grain soft bread e.g., rye bun, whole grain bread, or kavring ("Grovt mjukt bröd t.ex. rågbulle, fullkornsbröd eller kavring") | Background for calculation   - The most commonly bought whole grain breads are "Lingongrova 500g Pågen" and "Grötbröd 780g Pågen" - 1 "Lingongrova 500g Pågen" = 36 grams - 1 "Grötbröd 780g Pågen" = 56 grams   Assumptions   - 1 portion ≈ 40 grams - Sales from ICA Kvantum Stormarknad Luleå is representative for the study population |  |
| Crispbread | Crispbread ("Hårt bröd") | Background for calculation   - The most commonly bought crisp breads are "Knäckebröd Falu Råg-Rut 470g Wasa", "Normalgräddat knäckebröd 200g Leksands Knäckebröd", and "Knäckebröd Husman 520g Wasa" - 1 "Knäckebröd Falu Råg-Rut 470g Wasa" = 11.5 grams - 1 "Knäckebröd Husman 520g Wasa” = 12.9 grams   Weighted for estimations^1^   - 1 "Normalgräddat knäckebröd 200g Leksands Knäckebröd" = 13 grams   Assumptions   - 1 portion ≈ 12.5 grams (mean) - Sales from ICA Kvantum Stormarknad Luleå is representative for the study population |  |
| White bread | White bread e.g., sandwich bread, Molasses loaf, or Swedish flatbread ("Vitt bröd t.ex. formbröd, limpa eller tunnbröd") | Background for calculation   - The most commonly bought white breads are "Skärgårdskaka Hönö 750g Pågen", "Pågenlimpan 900g Pågen", and "Vetekaka 24-p 900g Polarbröd" - 1 slice "Skärgårdskaka Hönö 750g Pågen" = 42 grams - 1 slice "Pågenlimpan 900g Pågen" = 43 grams - 1 slice "Vetekaka 24-p 900g Polarbröd" = 38 grams   Assumptions   - 1 portion ≈ 40 grams - Sales from ICA Kvantum Stormarknad Luleå is representative for the study population |  |
| Rusks, rice cakes, or digestive biscuits | Rusks, rice cakes, or digestive biscuits ("Skorpor, riskakor eller digestive") | Background for calculation   - Swedish Food Agency recommend avoidance of rice cakes the first six years of life - The most commonly bought rusk is "Kardemumma skorpor 240g Pågen" - 1 "Kardemumma skorpor 240g Pågen" = 13 grams   Weighted for estimations^1^   - 2 thin corn cakes "Friggs Chiafrö & Havssalt" = 12 grams - 1 "Digestive Original 400g Mc Vities" = 15 grams   Assumptions   - 1 portion ≈ 15 grams - A child eats corn cakes instead of rice cakes - Sales from ICA Kvantum Stormarknad Luleå is representative for the study population |  |
| **Total breakfast cereals** | *Created variable based on quantified sub-types*   - Cornflakes or Special K - Musli or wholegrain cereals (e.g., All bran) - Oat pillows or Cheerios - Sweet cereals (e.g., Frosties) - Other breakfast cereals | Weighted for estimations^1^   - 1 dl “Frebaco Frukt & Bär müsli” = 50 grams | Note the small sample size of each subtype. Consider the power if subtypes are to be used instead of this total variable. |
| Cornflakes or Special K | Cornflakes or Special K | Background for calculation   - 1 portion “Frukostflingor majs osötad berikad typ cornflakes” = 30 grams (DietistNet)   Assumptions   - The subtypes are consumed equally frequently if several cereals were reported |  |
| Musli or wholegrain cereals (e.g., All bran) | Muesli or whole grain cereals e.g., All Bran | Background for calculation   - 1 portion “Frukostflingor müsli fullkorn m. bär” = 40 grams (DietistNet)   Assumptions   - The subtypes are consumed equally frequently if several cereals were reported |  |
| Oat pillows or Cheerios | Oat pillows ("Havrefras/kuddar") or Cheerios | Background for calculation   - 1 portion “Havrefras Original” = 30 grams (producer)   Assumptions   - The subtypes are consumed equally frequently if several cereals were reported |  |
| Sweet cereals (e.g., Frosties) | Sweet cereals e.g., Frosties, Honey Monster Puffs ("Kalaspuffar"), Coco Pops | Background for calculation   - 1 portion “Kalaspuffar” = 30 grams (producer)   Assumptions   - The subtypes are consumed equally frequently if several cereals were reported |  |
| Other breakfast cereals | Other breakfast cereals | Assumptions   - Unspecified cereals (“Andra flingor”) has a portion size of = 32.5 grams (mean of the other subtypes) |  |
| **Total fruit and berries** | *Created food group*   - Banana - Apple - Orange - Berries - Kiwi - Grapes - Other fruit - Juice - Jam or applesauce - Marmalade - Sweetened fruit soup |  | With juice and sweetened food items containing fruit or berries. |
| Jam or applesauce | Jam or applesauce | Background for calculation   - 1 portion “Jordgubbssylt” = 20 grams (DietistNet)   Weighted for estimations^1^   - 1 tsp = 6 grams - 1 tbsp = 16 grams   Assumptions   - 1 portion ≈ 20 grams |  |
| Sweetened fruit soup | Sweetened fruit soups such as hip rose soup or bilberry soup | Assumptions:   - 1 portion fruit soup = 1 portion beverage (i.e., 200 grams) | Traditional Swedish cuisine, often sweetened with sugar and made of either hip rose or bilberries which are boiled in water and thickened with starch. |
| Total fruit and berries (without juice) | *Created variable based on quantified sub-types*  Fruit or berries   - Banana - Apple - Orange - Berries - Kiwi - Grapes - Other fruit |  | With fresh fruit and berries, not including juice, jam or other sweetened varieties. |
| Banana | Banana | Background for calculation   - 1 peeled banana = 105 grams (DietistNet)   Assumptions   - The subtypes are consumed equally frequently if several fruits were reported |  |
| Apple | Apple or pear | Background for calculation   - 1 “medelstort Äpple m. skal” = 125 grams (DietistNet)   Assumptions   - The subtypes are consumed equally frequently if several fruits were reported |  |
| Orange | Citrus e.g., orange or clementine | Background for calculation   - 1 peeled “medelstor Apelsin” = 125 grams (DietistNet)   Assumptions:   - The subtypes are consumed equally frequently if several fruits were reported |  |
| Berries | Berries e.g., bilberries, raspberries or strawberries | Background for calculation   - 1 portion of strawberries = 125 grams (≈ 14 strawberries or 31 raspberries) (DietistNet) - Even in the squeeze pouches marketed as berry flavor, pouches solely with berries have not been found (i.e., the weight of a squeeze pouch cannot be translated to the weight of berries since it also contains fruits) - E.g., “Semper Sommar Smoothie Jordgubb 90 g” = 80% apple and 20% strawberry ≈ 18 grams berries   Assumptions:   - The subtypes are consumed equally frequently if several fruits were reported - 1 portion = ½ adult portion = 62.5 grams |  |
| Kiwi | Kiwi | Background for calculation   - 1 peeled “Grön kiwi” = 85 grams (DietistNet)   Assumptions:   - The subtypes are consumed equally frequently if several fruits were reported |  |
| Grapes | Grapes | Background for calculation   - 1 portion “Vindruvor” = 125 grams (DietistNet) - 1 grape = 12 grams (DietistNet)   Assumptions   - The subtypes are consumed equally frequently if several fruits were reported |  |
| Other fruit | Other fruit | Assumptions   - The subtypes are consumed equally frequently if several fruits were reported - Unspecified fruit (“Annan frukt”) weigh the mean of all other subtypes = 105 grams |  |
| **Total sauce** | Condiments e.g., Ketchup or sauce | Assumptions:   - The subtypes are consumed equally frequently if several sauces were reported |  |
| Vinaigrette | Vinaigrette (oil and vinegar) | Weighted for estimations^1^   - 1 tsp "Rapsolja, Zeta" = 4 grams   Assumptions:   - 1 portion = 1-2 tsp = 6 grams |  |
| Ketchup | Ketchup, tomato sauce or tomato salsa | Weighted for estimations^1^   - 1 tbsp "Ketchup, Felix osötad" = 16 grams   Assumptions:   - 1 portion = 1 tbsp ≈ 15 grams |  |
| Crème fraiche | Cream sauce, Crème fraiche or sour cream sauce ("Gräddsås eller crème fraiche/gräddfilssås") | Weighted for estimations^1^   - 1 tbsp "Créme fraiche, lätt, Arla" = 16 grams   Assumptions   - 1 portion = 1 tbsp ≈ 15 grams |  |
| Bearnaise | Bearnaise sauce or hamburger dressing | Weighted for estimations^1^   - 1 tbsp "Klassisk Bearnaise, Garant" = 16 grams   Assumptions:   - 1 portion = 1 tbsp ≈ 15 grams |  |
| Other sauce | Other sauce | Assumptions   - “Other sauce” weighs the mean of the other subtypes = 12.75 grams |  |
| **Pancake** | Pancakes, Swedish pancakes ("plättar"), oven baked pancakes, or waffles | Background for calculation   - 1 "Pannkaka tunn hemlagad" = 70 grams (DietistNet) - 1 portion "Pannkaka tunn hemlagad" = 225 grams (DietistNet) - 1 portion "Ugnspannkaka mellamjölk” = 225 grams (DietistNet)   Assumptions   - A child eats two pancakes per portion = 140 grams |  |
| **Pizza** | Pizza, pan pizza, pie, or pasty ("Pirog") | Background for calculation   - 1 portion “Pizza Capricciosa m. rökt skinka champinjon restaurang” = 275 grams (DietistNet) - 275 grams “Pizza Capricciosa m. rökt skinka champinjon restaurang” = 735 kcal   Assumptions   - A whole pizza contains >735 kcal - 275 grams pizza = ½ pizza - A child eats plainer pizzas (e.g., Vesuvio, Capricciosa, or Hawaii) and not more dense ones (e.g., Kebab) - A child eats ¼ of a pizza - ¼ pizza ≈ 135 grams |  |
| **Lasagna** | Lasagna, moussaka, or tortellini with meat | Background for calculation   - 1 can of Semper ready-made meals from eight months ≈ 190 grams (producer) - 1 can of Semper ready-made Lasagna/Moussaka from twelve months = 235 grams (producer) - When choosing portion size for "Potatis, ris, pasta eller liknande" a majority took the alternative corresponding to 75 grams - When choosing portion size for "Kött, fisk eller motsvarande vegetariska alternativ" the most common choices were 58 and 92 grams (48% and 37%, respectively)   Assumptions   - Lasagna is a mix of pasta and meat, and the portion size is the mean of the most reported portion sizes for the different food groups - 1 portion ≈ 75 grams |  |
| **Dairy products** | *Created food group*   - Cow’s milk - Yoghurt - Messmör (whey cheese) - Cheese - Crème fraiche |  | Probiotic drinks are not included in dairy since it could be e.g., proviva (juice based) and not milk based |
| Cow’s milk | Milk in a glass or a plate | Assumptions:   - 1 glass = 200 grams |  |
| **Total meat** | *Created food group*   - Sausage - Ham - Liver pâte - Black pudding - Meat dish - Hamburger - Minced meat - Chicken |  |  |
| Cured meat | *Created food group*   - Sausage - Ham - Liver pâte - Black pudding |  | Might be referred to as processed meat. The group is based on products commonly containing nitrites. |
| Red meat | *Created food group*   - Sausage - Ham - Liver pâte - Black pudding - Meat dish - Hamburger - Minced meat |  | A variable with all meat except the lean ones (poultry). |
| Offal | *Created food group*   - Liver pâte - Black pudding |  |  |
| Black pudding | Black pudding ("Blodpudding") |  |  |
| Sausage | Sausage dishes e.g., fried, boiled, gratinated or in a stew |  |  |
| Meat dish | Meat dishes e.g., roast, stew or wok |  |  |
| Chicken | Chicken or turkey e.g., grilled, bites, wok or in a stew |  |  |
| Hamburger | Hamburgers with bread or Tex-Mex such as tacos |  |  |
| Minced meat | Minced meat dishes e.g., Bolognese, lasagna or meatballs |  |  |
| **Total root vegetables** | *Created food group*   - Parsnip, Swedish turnip or celeriac - Roots - Carrot - Boiled or mashed potato - Onion |  | Note that potato in the form of French fries is not included. |
| Total without onion | *Created food group*   - Parsnip, Swedish turnip or celeriac - Roots - Carrot - Boiled or mashed potato |  | Excluding onion. |
| Total without onion and potato | *Created food group*   - Parsnip, Swedish turnip or celeriac - Roots - Carrot |  | Excluding potatoes and onion. |

| **Supplementary Table 2**. Energy needs and intakes of all children in the NICE cohort (N=523). | | | | |
| --- | --- | --- | --- | --- |
| **Variable** | **N** | **Mean (SD)** | **Median (IQR)** | **Min-max** |
| **All children (N=523)** |  |  |  |  |
| Energy intake (kcal) | 523 | 776.27 (306.27) | 753.55 (293.48) | 51.21-3044.16 |
| Average Energy Requirement | 520 | 800.87 (100.53) | 795.89 (169.69) | 477.53-1127.63 |
| Resting Energy Expenditure | 516 | 557.84 (59.41) | 551.68 (79.68) | 323.41-840.68 |
| Food Intake Level | 516 | 1.4 (0.55) | 1.35 (0.53) | 0.11-5.32 |
| Height (m) | 516 | 0.76 (0.04) | 0.76 (0.04) | 0.48-1.05 |
| Weight (kg) | 520 | 10 (1.24) | 10 (2) | 6-14 |
| **Girls (N=278)** |  |  |  |  |
| Energy intake (kcal) | 278 | 740.38 (267.93) | 735.17 (287.54) | 124.6-2531.94 |
| Average Energy Requirement | 276 | 766.76 (91.32) | 795.89 (79.59) | 477.53-1034.66 |
| Resting Energy Expenditure | 274 | 533.91 (47.87) | 536.7 (63.72) | 366.1-681.72 |
| Food Intake Level | 274 | 1.4 (0.52) | 1.38 (0.53) | 0.25-5.17 |
| Height (m) | 274 | 0.75 (0.03) | 0.75 (0.04) | 0.66-0.87 |
| Weight (kg) | 276 | 9.63 (1.15) | 10 (1) | 6-13 |
| **Boys (N=245)** |  |  |  |  |
| Energy intake (kcal) | 245 | 816.99 (340.65) | 776.26 (279.48) | 51.21-3044.16 |
| Average Energy Requirement | 244 | 839.45 (96.6) | 805.45 (80.54) | 563.81-1127.63 |
| Resting Energy Expenditure | 242 | 584.93 (59.66) | 583.28 (79.68) | 323.41-840.68 |
| Food Intake Level | 242 | 1.4 (0.58) | 1.31 (0.5) | 0.11-5.32 |
| Height (m) | 242 | 0.77 (0.04) | 0.77 (0.04) | 0.48-1.05 |
| Weight (kg) | 244 | 10.42 (1.2) | 10 (1) | 7-14 |

| **Supplementary Table 3**. Food intake (grams/day) of all 1-year-old children in the NICE cohort. | | | | |
| --- | --- | --- | --- | --- |
| **Food item** | **N** | **Mean (SD)** | **Median (IQR)** | **Min-max** |
| **Bulgur, couscous or quinoa** | 523 | 4.95 (10.74) | 0 (5.36) | 0-110 |
| **Rice** | 523 | 15.35 (14.63) | 16.07 (14.64) | 0-110 |
| **Pasta or noodles** | 523 | 32.02 (21.52) | 37.5 (21.43) | 0-176.79 |
| **Total potato** | 523 | 37.14 (26.01) | 37.5 (38.93) | 0-196.43 |
| French fries or fried potato | 523 | 4.9 (8.61) | 0 (5.36) | 0-86.43 |
| Boiled or mashed potato | 523 | 32.24 (23.93) | 31.43 (21.43) | 0-150 |
| **Vegetarian meat alternatives** | 523 | 6.46 (12.87) | 0 (6.57) | 0-92 |
| Beans or lentils | 523 | 1.21 (6.97) | 0 (0) | 0-92 |
| Soy products | 523 | 0.31 (2.46) | 0 (0) | 0-36.14 |
| Quorn | 523 | 0.29 (2.22) | 0 (0) | 0-24.1 |
| Roots | 523 | 0.83 (4.57) | 0 (0) | 0-46 |
| **Total seafood** | 523 | 18.25 (16.03) | 12.43 (13.14) | 0-99.79 |
| Seafood (without caviar) | 523 | 18.20 (16.04) | 12.43 (13.14) | 0-99.79 |
| Lean Fish | 523 | 9.31 (11.1) | 6.57 (13.14) | 0-92 |
| Fish fingers | 523 | 1.91 (4.6) | 0 (0) | 0-46 |
| Cod or pollock | 523 | 6.82 (9.53) | 4.14 (9.86) | 0-92 |
| Tuna | 523 | 0.58 (2.58) | 0 (0) | 0-24.1 |
| Fatty fish | 523 | 6.7 (8.51) | 4.93 (9.86) | 0-63.5 |
| Shellfish | 523 | 0.8 (2.79) | 0 (0) | 0-33.26 |
| Other fish | 523 | 0.43 (2.17) | 0 (0) | 0-23 |
| **Total vegetables** | 523 | 37.41 (42.39) | 23.57 (52.5) | 0-270 |
| Tomato or bell pepper | 523 | 4.2 (6.68) | 1.67 (6.43) | 0-67.5 |
| Cucumber or lettuce | 523 | 3.37 (6.07) | 0.8 (4.5) | 0-67.5 |
| Carrot | 523 | 5.77 (8.35) | 3 (7.86) | 0-90 |
| Onion | 523 | 2.26 (4.54) | 0 (2.5) | 0-30 |
| Corn | 523 | 5.1 (7.35) | 2.5 (7.5) | 0-67.5 |
| Avocado | 523 | 2.05 (4.74) | 0 (1.68) | 0-36 |
| Cauliflower or cabbage | 523 | 1.57 (4.84) | 0 (0) | 0-40 |
| Broccoli | 523 | 3.93 (5.91) | 1.07 (6) | 0-36 |
| Spinach | 523 | 0.27 (1.68) | 0 (0) | 0-22.5 |
| Green peas | 523 | 4.43 (6.87) | 1.5 (6.67) | 0-60 |
| Beans, lentils or chickpeas | 523 | 1.57 (4.1) | 0 (0) | 0-40 |
| Parsnip, Swedish turnip or celeriac | 523 | 2.21 (6.51) | 0 (0) | 0-90 |
| Pickled vegetables | 523 | 0.11 (1.09) | 0 (0) | 0-16.36 |
| Other vegetables | 523 | 0.43 (2.39) | 0 (0) | 0-24 |
| **Water** | 523 | 519.12 (150.43) | 600 (200) | 0-600 |
| **Sugary drinks** | 523 | 10.19 (53.8) | 0 (0) | 0-642.86 |
| Total soda | 523 | 5 (40.42) | 0 (0) | 0-600 |
| Sugar | 523 | 2.21 (30.34) | 0 (0) | 0-600 |
| Artificial sweeteners | 523 | 1.2 (16.71) | 0 (0) | 0-300 |
| Juice | 523 | 5.19 (27.8) | 0 (0) | 0-400 |
| **Plant-based dairy alternatives** | 523 | 11.85 (56.94) | 0 (0) | 0-600 |
| Oat drink | 523 | 8.9 (50.62) | 0 (0) | 0-600 |
| Soy drink | 523 | 0.1 (2.19) | 0 (0) | 0-50 |
| Sesame drink | 523 | 0.1 (2.19) | 0 (0) | 0-50 |
| Coconut milk | 523 | 1.17 (16.14) | 0 (0) | 0-300 |
| **Yoghurt** | 523 | 58.05 (85.15) | 14.29 (128.57) | 0-600 |
| Plain | 523 | 30.88 (72.26) | 0 (0) | 0-600 |
| Flavored | 523 | 13.17 (50.24) | 0 (0) | 0-400 |
| Other yoghurt | 523 | 3.39 (23.1) | 0 (0) | 0-400 |
| **Soup** | 523 | 6.01 (13.81) | 0 (14.29) | 0-100 |
| **Total gruel (“välling”)** | 523 | 218.18 (210.87) | 225 (450) | 0-675 |
| Mild whole grain | 523 | 90.86 (165.52) | 0 (112.5) | 0-675 |
| Whole grain | 523 | 88.08 (157.84) | 0 (128.57) | 0-675 |
| Corn gruel or gluten-free | 523 | 10.18 (56.08) | 0 (0) | 0-675 |
| Other gruel | 523 | 26.98 (103.25) | 0 (0) | 0-675 |
| **Porridge or rice pudding** | 523 | 146.81 (113.57) | 130 (176.43) | 0-450 |
| Oatmeal | 523 | 45.63 (85.14) | 0 (64.29) | 0-400 |
| Semolina or rice | 523 | 9.28 (37.15) | 0 (0) | 0-450 |
| Snack e.g., rice pudding | 523 | 0 (0) | 0 (0) | 0-0 |
| Powder-based for child | 523 | 83.79 (93.19) | 65 (130) | 0-390 |
| Other porridge | 523 | 8.11 (38.65) | 0 (0) | 0-365 |
| **Nuts** | 523 | 0.18 (1.07) | 0 (0) | 0-15 |
| **Raisins** | 523 | 2.75 (9.08) | 0 (0) | 0-84 |
| **Egg** | 523 | 9.28 (13.44) | 3.57 (10.71) | 0-100 |
| **Bread and crackers** | 523 | 31.25 (34.58) | 20 (38.08) | 0-180 |
| Total bread | 523 | 30.33 (34.05) | 20 (35.71) | 0-180 |
| Total wholegrain bread | 523 | 9.29 (18.57) | 0 (12.86) | 0-124.02 |
| Soft wholegrain bread | 523 | 8.38 (17.77) | 0 (12.86) | 0-120 |
| Crispbread | 523 | 0.91 (3) | 0 (0) | 0-18.75 |
| White bread | 523 | 21.03 (25.5) | 12.86 (38.57) | 0-120 |
| Rusks, rice cakes, or digestive biscuits | 523 | 0.92 (3.3) | 0 (0) | 0-30 |
| **Sandwich toppings** |  |  |  |  |
| Cheese | 523 | 3.63 (5.95) | 0 (5) | 0-45 |
| Ham | 523 | 1.78 (4.19) | 0 (1.61) | 0-45 |
| Marmalade | 523 | 0.01 (0.26) | 0 (0) | 0-6 |
| Liver pâte | 523 | 2.19 (4.67) | 0 (2.41) | 0-22.5 |
| Caviar | 523 | 0.05 (0.5) | 0 (0) | 0-7.5 |
| Messmör (whey cheese) | 523 | 0.12 (0.87) | 0 (0) | 0-9 |
| Tomato | 523 | 0.92 (3.93) | 0 (0) | 0-30 |
| Cucumber | 523 | 1.84 (5.96) | 0 (0) | 0-45 |
| **Total breakfast cereals** | 523 | 1.4 (6.82) | 0 (0) | 0-65 |
| Cornflakes or Special K | 523 | 0.14 (1.6) | 0 (0) | 0-30 |
| Musli or wholegrain cereals (e.g., All bran) | 523 | 0.37 (3.28) | 0 (0) | 0-40 |
| Oat pillows or Cheerios | 523 | 0.29 (2.65) | 0 (0) | 0-30 |
| Sweet cereals (e.g., Frosties) | 523 | 0.13 (2.64) | 0 (0) | 0-60 |
| Other breakfast cereals | 523 | 0.47 (4.1) | 0 (0) | 0-65 |
| **Total fruit and berries** | 523 | 164.6 (101.95) | 195 (125.68) | 0-960 |
| Jam or applesauce | 523 | 1.63 (5.1) | 0 (0) | 0-40 |
| Sweetened fruit soup | 523 | 3.41 (22.4) | 0 (0) | 0-200 |
| Total fruit and berries (without juice) | 523 | 154.35 (92.29) | 187.5 (116.68) | 0-360 |
| Banana | 523 | 46.96 (37.83) | 42 (49) | 0-210 |
| Apple | 523 | 43.3 (39.59) | 41.67 (62.5) | 0-187.5 |
| Orange | 523 | 21.71 (32.02) | 0 (41.67) | 0-125 |
| Berries | 523 | 17.94 (18.94) | 15.63 (31.25) | 0-125 |
| Kiwi | 523 | 6.97 (16.33) | 0 (0) | 0-85 |
| Grapes | 523 | 7.47 (18.43) | 0 (0) | 0-93.75 |
| Other fruit | 523 | 9.99 (26.02) | 0 (0) | 0-315 |
| **Total sauce** | 523 | 1.03 (3.07) | 0 (0) | 0-15 |
| Vinaigrette | 523 | 0.01 (0.13) | 0 (0) | 0-2.36 |
| Ketchup | 523 | 0.24 (1.08) | 0 (0) | 0-7.5 |
| Creme fraiche | 523 | 0.58 (2.05) | 0 (0) | 0-15 |
| Bearnaise | 523 | 0.09 (0.64) | 0 (0) | 0-5.89 |
| Other sauce | 523 | 0.1 (0.76) | 0 (0) | 0-10.02 |
| **Pancake** | 523 | 9.46 (11.42) | 10 (10) | 0-70 |
| **Pizza** | 523 | 3.04 (6.44) | 0 (9.64) | 0-67.5 |
| **Lasagna** | 523 | 7.18 (10.01) | 5.36 (16.07) | 0-58.93 |
| **Dairy products^1^** | 523 | 116.08 (157.34) | 44.46 (168.22) | 0-1026.43 |
| Cow’s milk | 523 | 53.7 (115.1) | 0 (42.86) | 0-600 |
| **Total meat** | 523 | 69.88 (46.1) | 59.14 (52.75) | 0-290.29 |
| Cured meat | 523 | 20.49 (22.12) | 16.57 (24.52) | 0-205.5 |
| Red meat | 523 | 55.57 (39.18) | 46.25 (43.29) | 0-253.64 |
| Offal^2^ | 523 | 6.17 (9.35) | 2.5 (8.31) | 0-71 |
| Black pudding | 523 | 3.98 (6.93) | 0 (6.57) | 0-63.5 |
| Sausage | 523 | 12.53 (15.13) | 12.43 (19.71) | 0-127 |
| Meat dish | 523 | 10.1 (14.55) | 4.14 (12.43) | 0-127 |
| Chicken | 523 | 14.31 (14.08) | 12.43 (15.57) | 0-99.79 |
| Hamburger | 523 | 2.63 (5.43) | 0 (4.14) | 0-72.29 |
| Minced meat | 523 | 22.35 (18.12) | 19.71 (16.57) | 0-127 |
| **Total root vegetables** | 523 | 43.31 (33.61) | 39 (42.05) | 0-200 |
| Total without onion | 523 | 41.05 (31.81) | 37.5 (39.37) | 0-200 |
| Total without onion and potato | 523 | 8.81 (14.49) | 3.93 (10.71) | 0-180 |
| **Buns, biscuits, or cookies** | 523 | 0.94 (3.21) | 0 (1.21) | 0-51 |
| **Candy** | 523 | 0.11 (0.81) | 0 (0) | 0-12.86 |
| **Chips** | 523 | 0.01 (0.15) | 0 (0) | 0-1.71 |
| **Ice cream** | 523 | 0.19 (1.06) | 0 (0) | 0-11.25 |
| **Probiotics** | 523 | 7.71 (29.33) | 0 (0) | 0-165 |

^1^ Includes reported intake of cow’s milk (in glass or plate), yoghurt, whey cheese (“Messmör”), Cheese, and Créme fraiche.

^2^ Includes liver paste and black pudding.

| **Supplementary Table 4.** Micro- and macronutrient intakes (per day) of all children in the NICE cohort (N=523). | | | | |
| --- | --- | --- | --- | --- |
| **Variable** | **Mean (SD)** | **Median (IQR)** | **Min-max** |  |
| Energy (kcal) | 776.27 (306.27) | 753.55 (293.48) | 51.21-3044.16 |  |
| Protein (g) | 31.57 (12.13) | 30.53 (13.59) | 2.24-121.27 |  |
| Fat (g) | 25.22 (12.33) | 23.71 (12.47) | 2.44-123.58 |  |
| Carbohydrates (g) | 100.13 (39.1) | 98.92 (36.05) | 4.62-404.37 |  |
| Fiber (g) | 9.42 (4.23) | 8.82 (4.74) | 0.94-40.67 |  |
| Salt (g) | 3.01 (1.4) | 2.79 (1.61) | 0.2-13.69 |  |
| Ash (g) | 8.37 (3.18) | 8.18 (3.25) | 0.5-29.41 |  |
| Water (g) | 1018.91 (276.27) | 1014.16 (319.7) | 37.9-2422.42 |  |
| Alcohol (g) | 0 (0) | 0 (0) | 0-0 |  |
| Monosaccharides (g) | 15.63 (8.47) | 14.57 (10.33) | 0.92-76.3 |  |
| Disaccharides (g) | 27.64 (13.99) | 26.52 (15.58) | 1.02-117.48 |  |
| Sucrose (g) | 10.07 (5.72) | 9.21 (4.82) | 0.73-70.92 |  |
| Whole grain (g) | 84.26 (57.32) | 75.62 (102.68) | 0.66-269.57 |  |
| Sum Saturated fatty acids (g) | 9.99 (4.76) | 9.55 (5.52) | 0.78-41.37 |  |
| Fatty acid 4:0-10:0 (g) | 0.54 (0.45) | 0.44 (0.57) | 0.01-3.23 |  |
| Fatty acid 12:0 (g) | 0.32 (0.27) | 0.27 (0.22) | 0.02-3.41 |  |
| Fatty acid 14:0 (g) | 0.88 (0.56) | 0.77 (0.68) | 0.06-4.37 |  |
| Fatty acid 16:0 (g) | 6.21 (2.69) | 6.13 (3.3) | 0.45-20.93 |  |
| Fatty acid 18:0 (g) | 1.79 (0.98) | 1.62 (1.08) | 0.13-9.19 |  |
| Fatty acid 20:0 (g) | 0.05 (0.04) | 0.04 (0.04) | 0-0.5 |  |
| Sum Monounsaturated fatty acids (g) | 9.4 (5.63) | 8.68 (4.52) | 0.89-72.77 |  |
| Fatty acid 16:1 (g) | 0.41 (0.23) | 0.38 (0.28) | 0.01-1.79 |  |
| Fatty acid 18:1 (g) | 8.71 (5.35) | 8.05 (4.14) | 0.83-70.69 |  |
| Sum Polyunsaturated fatty acids (g) | 3.86 (1.62) | 3.68 (1.86) | 0.4-13.85 |  |
| Fatty acid 18:2 (g) | 3.19 (1.39) | 3.06 (1.63) | 0.34-12.29 |  |
| Fatty acid 20:4 (g) | 0.04 (0.02) | 0.04 (0.03) | 0-0.16 |  |
| Fatty acid 18:3 (g) | 0.38 (0.21) | 0.34 (0.21) | 0.03-1.5 |  |
| EPA (Fatty acid 20:5) (g) | 0.04 (0.04) | 0.03 (0.04) | 0-0.37 |  |
| DPA (Fatty acid 22:5) (g) | 0.02 (0.02) | 0.02 (0.02) | 0-0.2 |  |
| DHA (Fatty acid 22:6) (g) | 0.09 (0.08) | 0.07 (0.09) | 0-0.68 |  |
| Cholesterol (mg) | 91.37 (53.49) | 82.84 (62.32) | 0.92-451.51 |  |
| Vitamin A (ug) | 381.34 (178.99) | 365.05 (230.22) | 26.52-1147.84 |  |
| Retinol (ug) | 310.77 (162.7) | 298.33 (210.95) | 14.4-908.53 |  |
| Beta-Carotene (ug) | 724.45 (804.56) | 480.66 (751.25) | 7.08-7960.08 |  |
| Vitamin D (ug) | 7.11 (3.76) | 7.38 (5.04) | 0.17-22.03 |  |
| Vitamin E (mg) | 7.45 (3.65) | 7.29 (5.16) | 0.46-25.48 |  |
| Vitamin K (ug) | 12.22 (10.28) | 9.16 (11.98) | 0.05-68.45 |  |
| Thiamine (mg) | 0.65 (0.25) | 0.64 (0.27) | 0.04-2.53 |  |
| Riboflavin (mg) | 0.77 (0.34) | 0.73 (0.37) | 0.05-2.99 |  |
| Vitamin C (mg) | 67.96 (33.2) | 68.65 (50.28) | 1.6-179.76 |  |
| Niacin (mg) | 6.8 (2.91) | 6.45 (3.27) | 0.38-24.09 |  |
| Niacin equivalents (mg) | 12.61 (4.99) | 12.3 (5.55) | 0.85-47.37 |  |
| Vitamin B6 (mg) | 0.85 (0.32) | 0.83 (0.35) | 0.07-3.07 |  |
| Vitamin B12 (ug) | 2.18 (1.08) | 2.03 (1.21) | 0.06-9.41 |  |
| Phosphorus (mg) | 660.08 (261.05) | 631.2 (287.26) | 37.39-2338.38 |  |
| Folate (ug) | 109.28 (50.93) | 101.33 (57.75) | 10.54-447.78 |  |
| Iodine (ug) | 48.67 (47.7) | 29.15 (54.34) | 0.27-352.11 |  |
| Waste (peel etc.) (%) | 26.88 (14.92) | 25.55 (18.52) | 0.18-116.19 |  |
| Iron (mg) | 8.66 (3.79) | 8.63 (5.31) | 0.37-22.22 |  |
| Calcium (mg) | 565.97 (277.13) | 550.36 (346.22) | 16.15-1973.82 |  |
| Potassium (mg) | 1323.52 (508.92) | 1271.81 (611.07) | 111.71-4285.4 |  |
| Magnesium (mg) | 133.23 (49.04) | 130.27 (50.92) | 8.54-421.61 |  |
| Sodium (mg) | 1190.92 (561.13) | 1105.94 (649.11) | 78.11-5461.88 |  |
| Selenium (ug) | 13.31 (6.56) | 12.56 (7.12) | 0.68-65.03 |  |
| Zinc (mg) | 4.24 (1.54) | 4.11 (1.91) | 0.27-15.3 |  |
| Sugars (g) | 1.95 (4.17) | 0.02 (1.54) | 0-22.25 |  |
| Sum Trans fatty acids (g) | 0.16 (0.17) | 0.11 (0.19) | 0-1.23 |  |


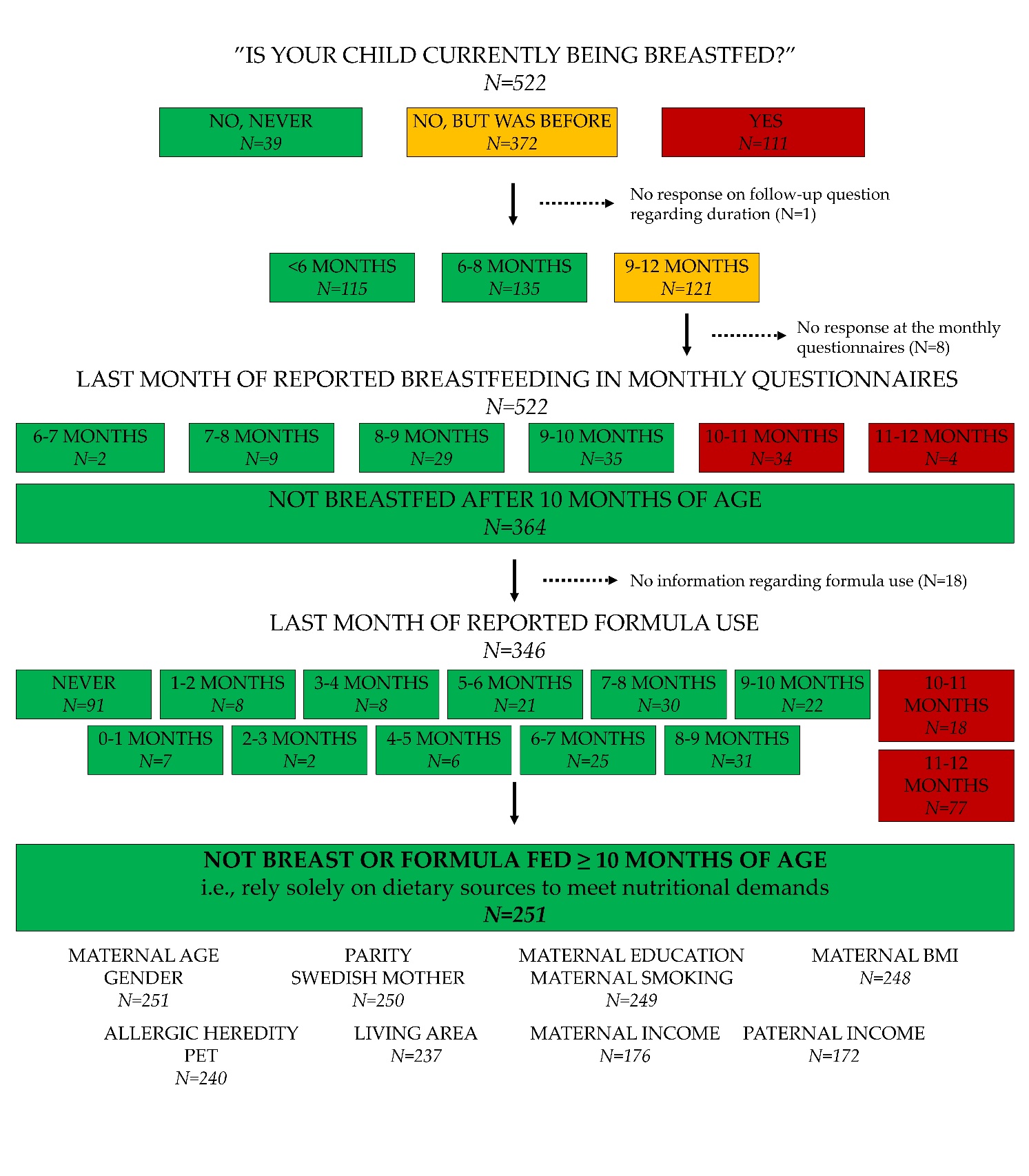


**Supplementary Figure 1.** Breastfeeding and formula use leading to exclusion from primary analyses.


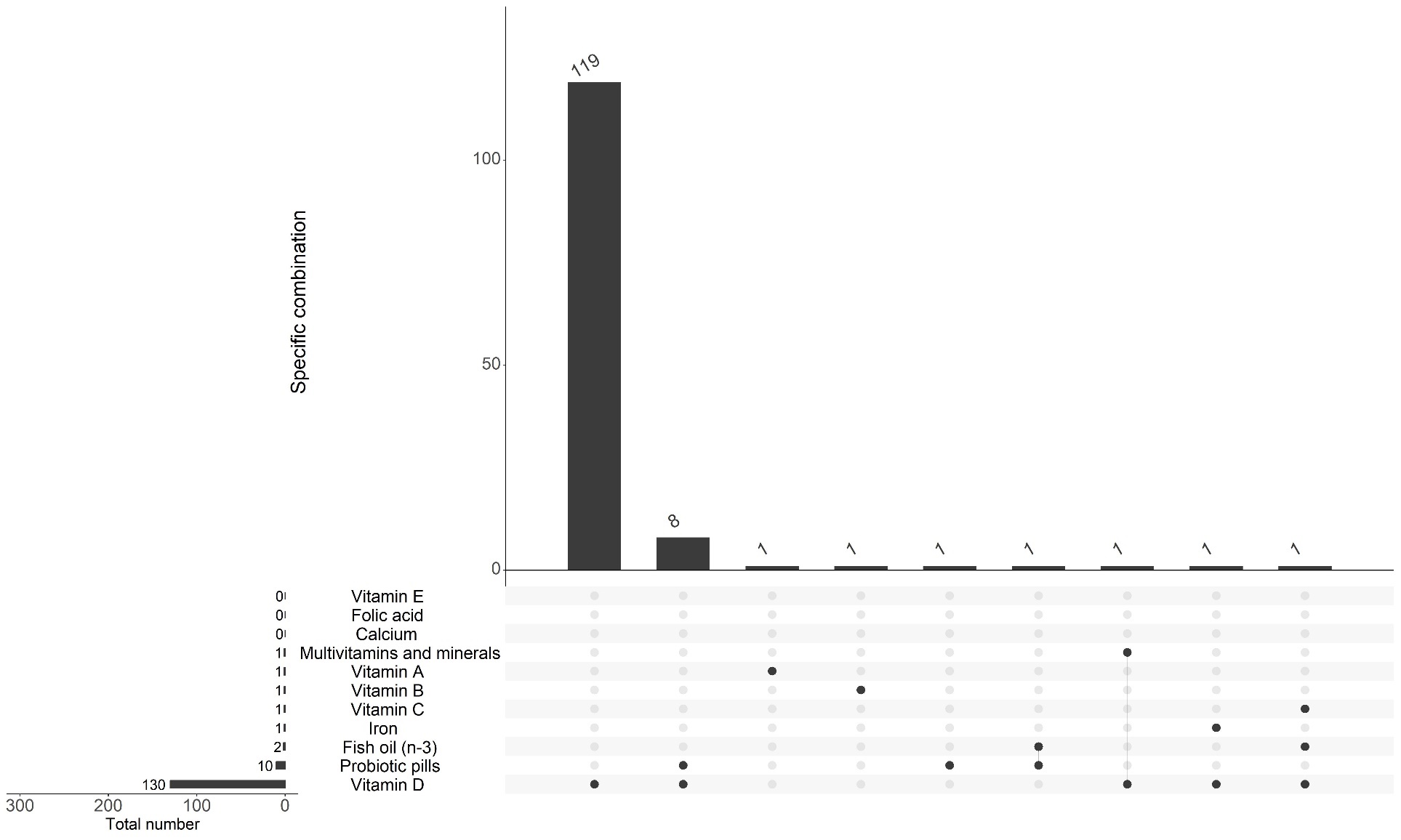


**Supplementary Figure 2.** Supplement use among the 251 children who did not receive breast milk or formula.

The main bar chart displays the number of children who received a specific combination of supplements as indicated below the chart with black dots. The bar chart in the bottom left specifies the total number of children receiving the supplement written on the same line to the right, regardless of other combinations.
